# Pigs, people, and proximity: a 6000-year isotopic record of pig management in Ireland

**DOI:** 10.1098/rsos.241300

**Published:** 2025-02-05

**Authors:** Eric Guiry, Fiona Beglane, Finbar McCormick, Eric Tourigny, Michael P. Richards

**Affiliations:** ^1^ Department of Anthropology, Trent University, 1600 West Bank Drive, Peterborough, Ontario, Canada K9L 0G2; ^2^ School of Archaeology and Ancient History, University of Leicester, Mayor’s Walk, Leicester LE1 7RH, UK; ^3^ Centre for Environmental Research Innovation and Sustainability, School of Science, Atlantic Technological University, Ash Lane, Sligo F91 YW50, Ireland; ^4^ School of Natural and Built Environment, University Road, Queen’s University Belfast, Belfast BT7 1NN, UK; ^5^ School of History, Classics and Archaeology, Newcastle University, Newcastle upon Tyne NE1 7RU, UK; ^6^ Department of Archaeology, Simon Fraser University, Education Building 9635, 8888 University Drive, Burnaby, British Columbia, Canada V5A 1S6

**Keywords:** archaeology, historical ecology, stable isotopes, animal proximity, woodland, pannage

## Abstract

The ways that pigs interact with humans are more flexible than other livestock. This plasticity means that pig behaviour can evidence a tremendous range of cultural phenomena, some of which may not otherwise show up in the archaeological record. We explore how people and pigs interacted in Ireland over 6000 years (4000 BC–AD 1900) from the perspective of isotopic zooarchaeology, using a large sample of pigs from 40 sites. Results demonstrate continuity and dramatic change. While pig diets show an emphasis on pannage throughout much of the period, husbandry was fundamentally reconstructed in the early medieval period. Through prehistory, pigs were herded in areas distant from human settlements, whereas later they were relocated to live near people. We explore potential implications of these patterns at a range of scales, from economics, to perspectives on zoonoses, and animal agency. While syntheses of a similar scope are needed for other areas of Europe, these findings may reflect a uniquely Irish trajectory of human–animal relationships.

## 1. Introduction

Pigs hold a special place in many cultures and regions of the world. We recognize their intelligence and social nature as standing apart from other livestock and this helped to create diverse ways of husbanding and otherwise relating to pigs [1]. Research on the ways in which pigs have been incorporated into human societies and landscapes through time can open valuable windows onto human activity in the past. Such work can, for instance, reveal clues about what resources were available for human societies as well as the decisions people made about both how to practice agriculture and how to situate other intelligent, agentive beings in their worlds [1,2]. In the context of archaeological interpretations, the complexity and broad scope of pig behaviour, from the perspective of both intelligence and dietary range, represent challenges and opportunities. On the one hand, their sheer flexibility (in terms of the diverse possible ways of raising pigs) means that evidence for specific behaviours and approaches to pig husbandry might be equivocal. On the other hand, these same qualities make pig husbandry something that could be moulded into unique sets of practices in different places and times, something which would be well positioned to signify cultural differences and meanings.

**Figure 1.**
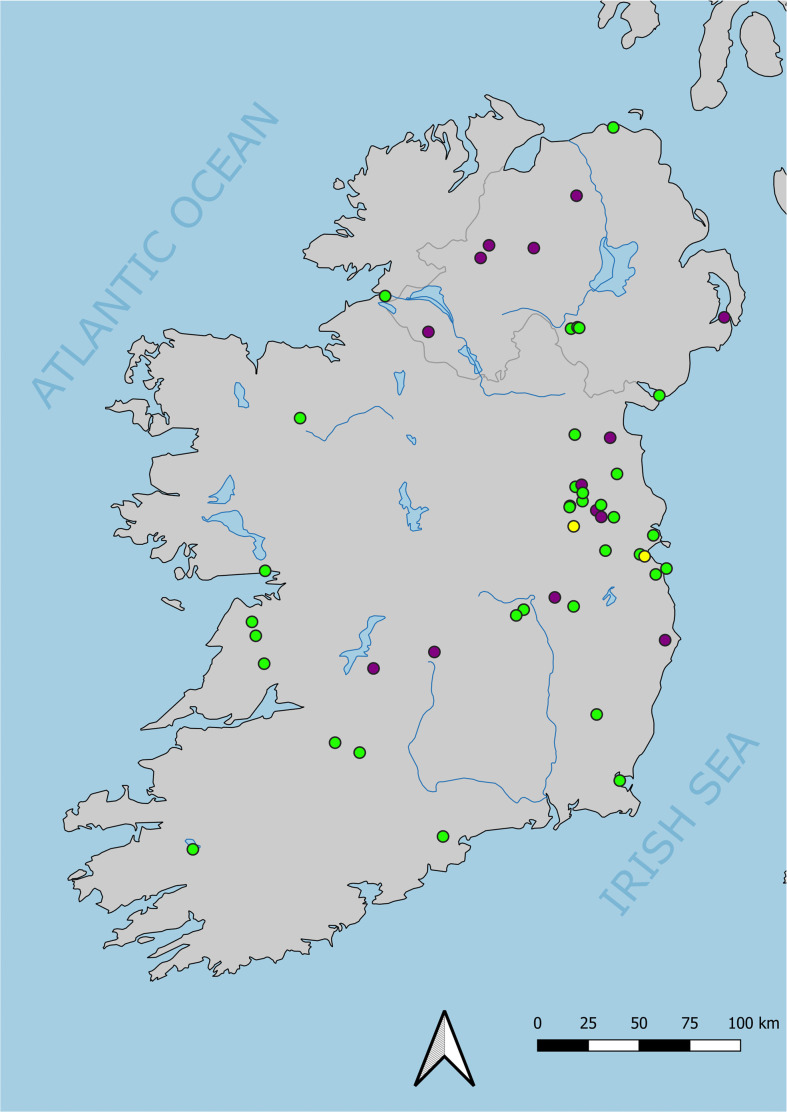
Map showing site locations. Symbols: yellow are sites with pigs only; green are sites with pigs and baseline data from cattle; purple are sites with baseline data from cattle only. See electronic supplementary material, figure S1, for version listing sites and electronic supplementary material S2 for version showing sites by time period.

In this paper, we explore the ways in which pigs have been husbanded at a macro-scale across Ireland over 6000 years (*ca* 4000 BC to AD 1900, including 40 sites; figure 1) within a dietary framework structured by isotopic compositions of archaeological pig and cattle bones. Documenting what pigs ate at different times offers perspective on continuity and change in Irish human–animal relationships that could not easily be achieved using other lines of archaeological evidence or through smaller-scale, site-level approaches. These data establish an interpretive context in which, at a coarse scale, we can observe patterns in the degree to which pigs lived near people. We explore both of these narratives (i.e. diet continuity and proximity to people) as evidence for what may have been a distinctly Irish trajectory of human–animal relationships. We also highlight some of the implications these perspectives have for other research areas, including for understanding trends in potential for zoonoses, the structuring of connections between styles of animal husbandry and settlement patterns in late prehistoric Europe, and approaches to comprehending animals as persons.

## 2. Context

### 2.1. Pig ecology

The domestic pig (*Sus scrofa domesticus*) is closely related to the wild pig (*Sus scrofa*), which is often termed ‘wild boar’, the phrase used here, although a boar is technically the male of either taxon. The wild and domestic forms can freely interbreed. Zooarchaeologically the two are differentiated mainly by the size of bones and teeth, with wild boar being substantially larger than prehistoric and historic domesticates [3,4]. Wild boars maintain an ecological niche that overlaps with that of humans. They are predominantly herbivorous, consuming a range of plant materials including nuts, seeds, mast (acorns), roots, herbs, and fruit, which are high in carbohydrates. As omnivores, they will also consume carrion, young birds and mammals, and eggs of ground-nesting species [5], which would have the effect of increasing the protein and fat contents of the diet. Extensively herded, domestic pigs can be allowed to forage in woodland and may also, for example, glean grain in fields after harvesting. Alternatively, pigs can be kept intensively in sties and fed with a variety of foodstuffs including grain, as well as waste food from human settlements including vegetable trimmings, meat scraps, and dairy byproducts such as whey. Pigs can also be fed with human excrement, which is a source of nutrients including animal protein, although this has significant risks of parasite transfer [6]. Sty feeding can offer more animal-protein-rich diets than woodland feeding, and therefore typically promotes faster weight gain. Extensive and sty-based husbandry methods can also be combined on a seasonal basis [7]. Given these factors, extensively herded domestic pigs are likely to have similar isotopic signals (see below) to wild pigs, with differences from this likely to be caused by seasonal or year-round sty-based feeding.

Skeletally, pigs are fully grown at *ca* 42 months, based on fusion of the long bones [8]. Given adequate food, piglets from modern breeds initially grow quickly, before the growth rate tails off and a maximum weight is achieved at *ca* 1000 days (2.75 years) [9]. In modern pig husbandry, following a carefully controlled high-protein diet, the age at slaughter is typically *ca* 6 months. However, this was not the case archaeologically, with ages of 1.5–2.5 years more typical in Ireland [10]. In his ethnographic study of woodland-oriented pig rearing in 1960s Spain, Parsons [11] noted that for these extensively managed herds, food supplies varied through the year and growth was slow. In their first autumn, the juvenile pigs could not fatten on the mast crop, which was high in carbohydrate, but lacked the protein needed for skeletal development. Instead, the pigs were kept for another year, and based on a January farrowing the main period of slaughter was at 20–24 months, with these older pigs having been fattened on mast (acorns and other tree nuts) immediately prior to slaughter.

### 2.2. Pigs in Ireland’s past

While archaeological and historical evidence for human–pig relationships in Ireland is reviewed where relevant in §4, it is worth outlining some of the broader patterns evident in the literature. The richest body of evidence for early Irish pig husbandry has been assembled by Kelly [12] from law-texts, wisdom-texts, annals and tales recorded in the early medieval period (*ca* AD 600–800). Compared with the archaeological evidence from prehistory, these sources provide a highly detailed interpretive framework (see §4).

McCormick has synthesized the zooarchaeological evidence for prehistoric- and historic-period pigs in faunal assemblages from across Ireland (for reviews, see [13–20]). While these sources note considerable gaps in the available faunal record during the prehistoric period, generally cattle remains are more frequent than those of pigs [13]. Notably pig-rich assemblages occur at Late Neolithic Newgrange, Bronze Age Lough Gur, and Early Iron Age Navan Fort [13]. Interpreting evidence for human–pig interactions from these assemblages is challenging, however, because it is conceivable that they could reflect human activities, such as ceremonial feasting, considered special or unusual by their participants. In the context of this limited body of evidence, knowledge about the roles pigs played during the prehistoric period in Ireland remains limited [13,14].

By contrast, archaeological evidence for pigs becomes more abundant in the historical period, with evidence pointing to greater variation in the importance of pigs relative to other livestock across different site types and environments [14,17,20]. Combining documentary and archaeological evidence, we can see a more complete picture of typical management practices [12,17]. Pigs were single-farrowing (spring), with up to nine piglets per litter. Husbandry practices typically incorporated feed from both dairy products and pannage as well as grain-based resources (see §4). The peak age for pig slaughter was between the ages of 1.5 and 2.5 years at most sites, which likely optimized the quantity of meat produced relative to the feed input, though they tended to be killed at an earlier age in some urban contexts [14]. Male pigs were typically dispatched younger than females. While some pork was consumed fresh, much was cured by salting for later use [17].

### 2.3. Isotopic ecology

Stable carbon (*δ*^13^C) and nitrogen (*δ*^15^N) isotope composition of collagen extracted from archaeological bone can tell us what animals ate. Moreover, to the extent that the abundance of specific foods may differ across space and time, animals’ bone collagen *δ*^13^C and *δ*^15^N compositions can offer clues about where they lived. The core concept is that the ways in which carbon and nitrogen are sourced and cycled across environments create distinctive isotopic signatures in select foods, which are then passed on to their consumers. In turn, patterns in these signatures across animal bone assemblages can serve as indicators for environmental and cultural processes at a wide range of scales [21,22].

While we review key dimensions of these isotopic indicators as they become relevant throughout §4, it is worth outlining some of the main axes of isotopic variation in Ireland. As omnivores and opportunists, interpretation of pig isotopic compositions can be highly complex, and assessment of individual pig isotopic compositions will almost always come with caveats about potential for equivocality in interpretations. By contrast, when data from pigs are aggregated across larger site-, community- and regional-scale assemblages, robust patterns can be discerned.

Ireland is a C_3_-dominated environment, and hence *δ*^13^C variation is expected to occur across a relatively narrow spectrum [23], in contrast to the kinds of larger-scale isotopic differences expected from mixed consumption of both C_3_ and C_4_ plants [24]. As outlined in table 1, variables include the canopy effect (i.e. from feeding in denser woodlands [26]), consumption of photosynthetic (e.g. leaves) versus non-photosynthetic (e.g. mast, grain) plant tissues [29], trophic level [34] and use of aquatic resources including lacustrine, riverine and wetland habitats [37]. Given that trophic enrichment is small for *δ*^13^C (see §6) and that the vast majority of our sample comes from places where marine foods would not be abundant, we expect *δ*^13^C variation in our dataset to be governed by the canopy effect and consumption of non-photosynthetic plant foods (e.g. mast, grain).

**Table 1.** Some key axes of carbon isotope variation for Irish omnivores.

axis	*δ*^13^C	detailed description of axis in context of Ireland
denser canopy versus open air	↕	canopy cover can lead to lower *δ*^13^C in animals feeding near ground level in closed woodland areas relative to those feeding in open pasture lands [25]. Causes include: (1) slower air movement/exchange under canopies, which allows woodland foliage to incorporate more ^13^C-depleted CO_2_ (released from decomposing vegetation in soils) and (2) influence of lower light levels on understory foliage, which impacts ability to discriminate against ^13^C [26]. Based on a large sample of archaeological Irish cattle, *δ*^13^C variation associated with this open–closed canopy feeding spectrum spans *ca* <3‰ [23]
leaves versus nuts/seeds	↕	consumption of non-photosynthetic plant tissues such as mast (tree nuts) can lead to higher *δ*^13^C in animals feeding on pannage compared to those feeding on vegetation [27, 28]. This is caused by an isotopic fractionation that occurs as nutrients are remobilized in the process of forming different portions of plants’ tissues, such that non-photosynthetic structures (e.g. nuts, seeds) become ^13^C-enriched relative to photosynthetic structures (e.g. leaves, stems) [29]. While the isotopic compositions of mast are expected to vary based on a wide range of environmental factors, including the canopy effect [30], based on archaeological and contemporary observations, a spectrum of no-mast to intensive-mast feeding could result in a *δ*^13^C range of *ca* 4‰ [31, 32]. Cereals and grains should also result in similarly elevated *δ*^13^C
animal protein	↑	consumption of animal protein can lead to higher *δ*^13^C in omnivorous and carnivorous animals [33]. This is caused by processes occurring as amino acids are synthesized, processed, and assimilated from diet to consumer tissues, which, at the whole-protein level (i.e. collagen) selectively retain ^13^C-enriched building blocks. Compared to trophic enrichment for *δ*^15^N, this effect is small, and is on average thought to be *ca* 0.5‰ per trophic level for *δ*^13^C (though variation is known; see [34])
aquatic foods	↕	consumption of aquatic foods, including those from marine, lacustrine, riverine, and wetland habitats, can drive animal *δ*^13^C in both directions (for a review, see [35]). Plants growing in flooded areas draw CO_2_ from an isotopically homogeneous source (the atmosphere), but may show greater isotopic variation based on water availability, including lower *δ*^13^C due to waterlogging [36]. More variation can be expected for submerged primary production, which draws CO_2_ from the water column. These effects stem from differences in the way carbon is cycled in aquatic, relative to terrestrial, systems. Marine foods of animal or plant origin are generally thought to be ^13^C-enriched because the primary CO_2_ source in marine environment has more ^13^C than the source used by terrestrial plants [37]. By contrast, freshwater carbon sources can incorporate CO_2_ from a wide range of isotopically distinctive materials. This can include CO_2_ respired from breakdown of terrestrial vegetation, resulting in extremely low *δ*^13^C for terrestrial consumers of freshwater fish in some regions [38]. By contrast, carbon cycling in some areas can serve to increase baseline freshwater *δ*^13^C and to reduce primary producer discrimination against ^13^C, leading to high *δ*^13^C overlapping with most of the marine range [35]. Overall, a potential *δ*^13^C range for marine and freshwater foods in Ireland could be as wide as 30‰

Previous work by Hamilton *et al*. [39] has highlighted the potential relevance of fungus consumption for explaining variation observed in pig *δ*^13^C values, but we do not believe this offers a more realistic explanation compared to mast/grain feeding. Hamilton *et al*.’s [39] work proposing a potential importance of fungi for pig *δ*^13^C values was aimed at comprehending an apparent paradox in isotopic patterns observed across the British Neolithic and Iron Age. Namely, why pigs that, based on context, should have been consuming more mast in woodlands, habitats which are generally associated with low *δ*^13^C values (table 1), seem to have high *δ*^13^C values. What has since become clear is that mast consumption will be associated with higher, not lower, *δ*^13^C as it is a non-photosynthetic, and thus ^13^C-enriched, plant tissue (for a review, see [27]). In that context, the need for a woodland-associated source of ^13^C-enriched foods (i.e. fungi) to explain these paradoxical isotopic patterns is met by the very thing (mast) that was expected to be driving pig husbandry into woodlands.

Pig *δ*^15^N compositions are governed primarily by trophic position and baseline variation [40]. In contrast to *δ*^13^C, *δ*^15^N has a large trophic enrichment factor (TEF) meaning that it undergoes a substantial shift (*ca* +3.6‰) at each trophic step [34]. For this reason, pigs eating some animal protein (e.g. meat, dairy, or even human faeces) will have higher *δ*^15^N on average than their herbivorous counterparts. It also means that particularly young pigs that are still consuming, or have recently been weaned from sow’s milk will have elevated *δ*^15^N relative to adults in the same population [41]. As aquatic environments have additional trophic levels (i.e. carnivores that eat other carnivores), consumption of certain fish can also result in very high *δ*^15^N values [42]. In addition to these TEF-related variables, there is considerable potential for variation in baseline *δ*^15^N. Specifically, nitrogen cycling and sourcing processes at the plant–soil level, which are impacted by diverse human (e.g. manuring, stocking rates) and non-human phenomena (e.g. mycorrhizal changes), change the baseline starting point from which *δ*^15^N trophic enrichment occurs in both terrestrial and aquatic environments (for reviews, see [35,40]). Upward baseline shifts have been observed in Irish prehistory and are thought to reflect increasing human land management impacts [43]. It is therefore essential to consider the baseline when interpreting pig *δ*^15^N.

Lastly, it is worth highlighting that, due to the complexity of carbon and nitrogen sources and cycling in wetlands, animal husbandry practices that make systematic and sustained use of bogs, fens and marshes could contribute to increased isotopic variation for pigs and cattle in our dataset (for reviews, see [35,44]). This would particularly be the case for use of food webs that were based on submerged aquatic vegetation (e.g. phytoplankton, macroalgae etc.), which can produce foods with isotopic compositions conceivably spanning a range of >25‰ for both *δ*^13^C and *δ*^15^N across short distances within a single aquatic system [35]. This heightened potential for variation occurs because submerged aquatic vegetation must rely on carbon and nitrogen sourced from the water column, which is in turn governed by a varied range of (often spatially heterogeneous) cycling processes. Emergent aquatic vegetation, by contrast, sources carbon mainly from the atmosphere and will have much less *δ*^13^C variation (i.e. similar to terrestrial plants). Recent work has suggested that flooding may affect isotopic compositions of willow trees, potentially driving their *δ*^13^C upwards or downwards under conditions of shorter- and longer-term flooding (with extreme flooding altering foliar *δ*^13^C by as much as −1.2 ± 0.5‰ on average [36]). More work is needed, however, to establish whether this also occurs in plants that have evolved adaptations requiring freshwater wetland conditions. It is also worth pointing out that the anoxic conditions established by permanent waterlogging can influence the nitrogen cycling, causing emergent aquatic vegetation to have higher *δ*^15^N values [45]. While use of wetland resources from food webs based on either submerged or emergent aquatic vegetation will remain a potential source of variation, we do not expect that this will be a primary driver of variation in pig or cattle isotopic compositions in this study. This is because the broad scale, aggregating approach we take should obviate potential influences from occasional and more spatially localized instances of intensive wetland use.

### 2.4. Isotopic work on Irish pigs

Previous interpretations of archaeological pigs in Ireland have begun to sketch out a narrative for broader trends in husbandry. Most direct interpretations have focused on the medieval period and are limited in nature because the studies from which they were drawn were focused on, and used pigs as a baseline for interpreting human diet and mobility. Knudson *et al*. [46] noted that isotopic compositions of Hiberno-Norse pigs (*n* = 12) in County Dublin do not indicate marine protein consumption. Ryan *et al*. [47] noted that isotopic compositions from early medieval-period pigs (*n* = 7) in County Meath could reflect variation in life stage, location and animal-management practices. McKenzie *et al*. [48] noted that a later medieval pig from County Donegal had an isotopic composition that could reflect an omnivorous diet. Work by Madgwick *et al*. [49] and Guiry *et al*. [23] offer interpretations that are more animal-oriented and are therefore an exception to this human-focused trend. Madgwick *et al*. [49], examining 19 pigs from Iron Age Navan Fort in County Armagh, found that while pigs were mainly herbivorous, some may have been omnivorous. Exploring isotopic variation among 178 pigs and wild boar from sites across Ireland, spanning the later Holocene (including many from the samples used in this study), Guiry *et al*. [43] showed that pig *δ*^15^N increased through time across the island. While this trend was thought to reflect increasing baseline *δ*^15^N (i.e. at the plant–soil level), higher *δ*^15^N in pigs from later periods was recognized as evidence for consumption of animal protein. Building on this existing work, in this paper we interpret data from 238 pigs from 40 sites. While some of these data have been sourced from the literature, 88% of *δ*^13^C (*n* = 209) and 40% of *δ*^15^N (*n* = 93) values are new (see §3). We use the large body of archaeological herbivore data available from Ireland [23,43,48,49] to correct for baseline variation. Specifically, we use mean values from 344 cattle from 49 widely distributed sites (figure 1) aggregated by time period (and site, where sample sizes allow; see §6) to establish what herbivorous *δ*^15^N should look like. We then compare pigs to these baseline data to evaluate the extent to which they were herbivores or omnivores (see §6).

### 2.5. Hypotheses and research questions

Based on previous isotopic and zooarchaeological work, as well as information from the historical record, we can structure the following research questions and hypotheses.

1. To what extent did pannage and animal products feature in medieval pig diets?
  Hypothesis. If historical narratives indicating the dual importance of animal protein^1^ and mast for pig husbandry [12] are correct, we will see relatively high *δ*^15^N and *δ*^13^C values on average in pigs from that timeframe. This could include grain consumption, which, like mast, will result in elevated *δ*^13^C values relative to most other foods (i.e. foods derived from photosynthetic plant tissues or products of animals eating these foods). Existing isotopic evidence shows some support for the importance of animal protein in pig diets evidenced in the early documentary sources (especially dairy byproducts, such as whey) [43], though baseline variation has not been corrected for in past work, nor has the relevance of pannage and grain been considered.
2. Looking further back in time, to what extent does the dual-diet approach to pig husbandry (i.e. animal protein and pannage) that is featured in the early medieval period literature, characterize pig husbandry practices in earlier periods?
  Hypothesis. If pig husbandry in Ireland has always involved feeding pigs waste generated from human settlements and other activities and driving pigs to pannage, then we will see a similar pattern to that expected for the medieval period, involving higher *δ*^15^N and *δ*^13^C values relative to contemporaneous herbivorous livestock. While existing isotopic evidence suggests that at least some pigs were more herbivorous in the prehistoric timeframe, interpretation of relative omnivory has been limited by baseline variation. Moreover, the pool of published *δ*^13^C data available for prehistoric pigs has been too small to consider trends in pannage.

## 3. Results

**Figure 2.**
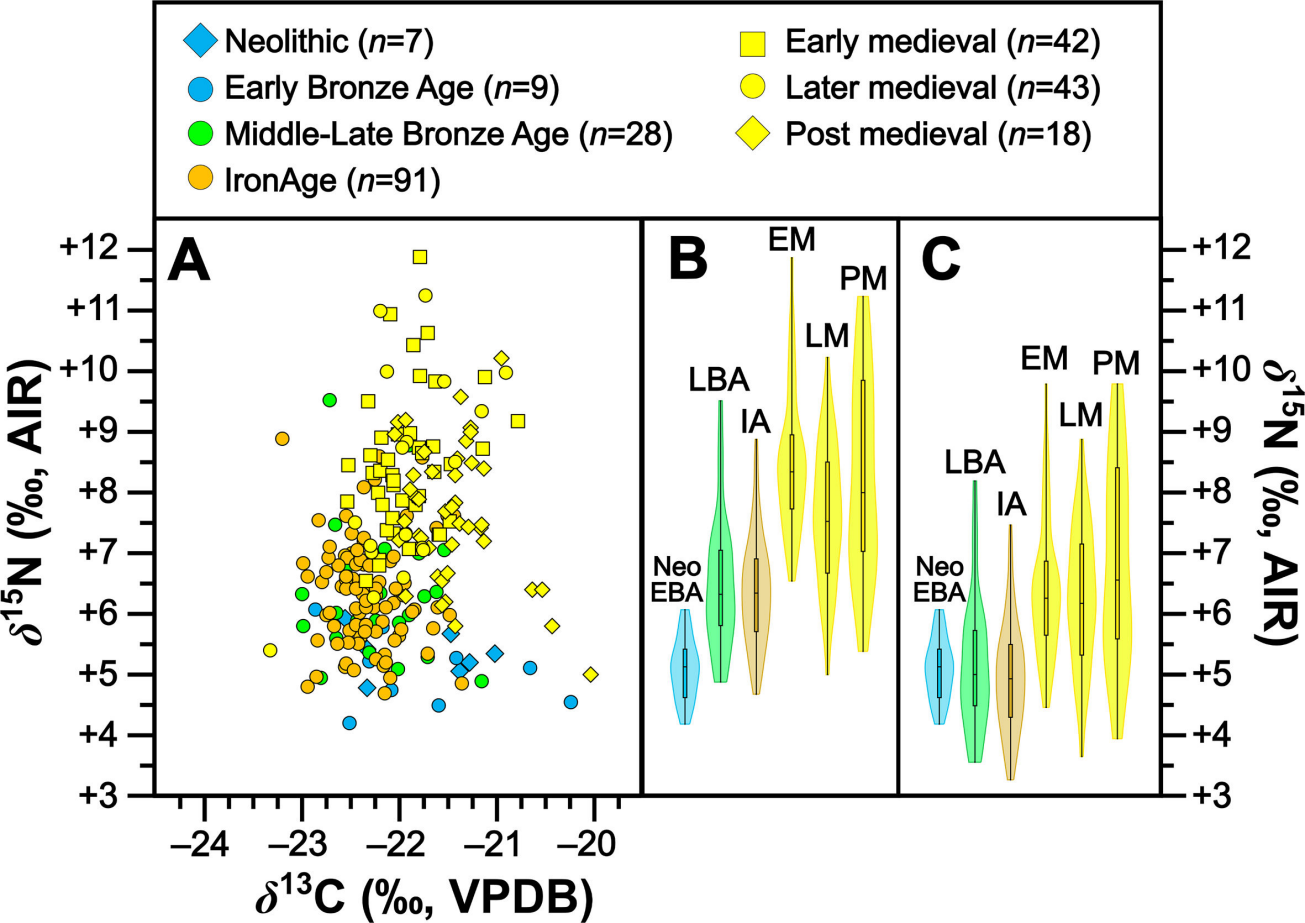
Isotopic compositions for pigs grouped by time period shown in bivariate space (*a*), with violin and box plots showing kernel density for *δ*^15^N for raw (*b*) and baseline-corrected (*c*) *δ*^15^N. For (*b*,*c*): Neo EBA = Neolithic and Early Bronze Age; LBA = Middle Late Bronze Age; IA = Iron Age; EM = early medieval; LM = later medieval; PM = post-medieval. For (*c*), baselines for all later periods have been corrected to the earliest (Neolithic) period (see §3 for description of methods).

We analysed 302 pig specimens from 38 sites, of which 209 samples from 34 sites produced isotopic compositions associated with viable quality control metrics (electronic supplementary material, table S6). All 209 *δ*^13^C values are new whereas 93 of these *δ*^15^N values are new and 116 are previously published [43]. Of the samples failing quality control, most (95%) were ultrafiltered rather than NaOH treated (see §6). We exclude a further four samples from interpretations based on issues of low chronological resolution or osteological indicators suggesting samples are from younger pigs, which could have *δ*^15^N values influenced by milk suckling [41]. To this we can add previously published isotopic compositions from 33 pigs from seven sites [46,48,49] giving a total of 238 pigs from 40 sites (all data listed in electronic supplementary material, table S6). Data are visualized in figure 2 and means and s.d. for all periods are shown in table 2.

**Table 2.** Means and s.d. for pigs as well as statistical and means comparisons of pigs with cattle from respective time periods and sites (where *n >* 5 for both cattle and pigs). For raw data and sources, see electronic supplementary material, table S6. Statistically significant differences are shown in bold font. For full statistical results, including normality and variances tests as well as means comparisons, see electronic supplementary material, table S7.

grouping	pig				cattle				*δ*^13^C comp.		*δ*^15^N comp.	
	*n* =	sites	*δ*^13^C (‰)	*δ*^15^N (‰)	*n* =	sites	*δ*^13^C (‰)	*δ*^15^N (‰)	pig–cattle	*p* =	pig–cattle	*p* =
*timeframe comparisons*												
Neolithic–Early Bronze Age	16	7	−21.7 ± 0.7	5.2 ± 0.5	36	13	−22.4 ± 0.6	5.1 ± 0.7	0.62	**0.001**	0.08	0.709
Neolithic–Early Bronze Age without boar	14	5	−21.6 ± 0.8	5.2 ± 0.5								—
Middle–Late Bronze Age	28	5	−22.2 ± 0.5	6.5 ± 1.1	70	12	−22.8 ± 0.5	6.3 ± 0.6	0.60	**<0.001**	0.21	**0.001**
Iron Age	91	6	−22.3 ± 0.4	6.4 ± 0.9	44	6	−22.4 ± 0.4	6.4 ± 0.9	0.19	**0.009**	−0.02	0.894
Middle–Late Bronze and Iron Age	119	11	−22.2 ± 0.4	6.4 ± 0.9	114	18	−22.7 ± 0.5	6.3 ± 0.8	0.44	**<0.001**	0.07	0.961
early medieval	42	6	−21.9 ± 0.4	8.5 ± 1.2	71	7	−22.2 ± 0.4	7.1 ± 1.0	0.29	**0.001**	1.42	**<0.001**
later medieval	43	10	−21.4 ± 0.4	7.6 ± 1.1	60	7	−22.4 ± 0.5	6.3 ± 1.3	1.01	**<0.001**	1.32	**<0.001**
post-medieval	18	8	−21.9 ± 0.5	8.3 ± 1.7	63	15	−22.4 ± 0.5	6.4 ± 1.1	0.49	**<0.001**	1.84	**<0.001**
medieval and post-medieval	103	20	−21.7 ± 0.5	8.1 ± 1.3	194	22	−22.3 ± 0.5	6.6 ± 1.2	0.63	**<0.001**	1.47	**<0.001**
*intra-site comparisons*												
Ross Island (Early Bronze Age)	5	—	−21.4 ± 1.0	5.0 ± 0.6	8	—	−22.3 ± 0.5	5.3 ± 0.5	0.95	**0.040**	−0.37	0.273
Haughey's Fort (Middle–Late Bronze Age)	14	—	−22.3 ± 0.5	5.9 ± 0.7	51	—	−22.9 ± 0.5	6.3 ± 0.7	0.59	**<0.001**	−0.40	0.062
Dún Ailinne (Iron Age)	15	—	−22.2 ± 0.4	6.3 ± 1.0	15	—	−22.2 ± 0.4	6.8 ± 0.9	0.02	0.897	−0.55	0.135
Navan Fort (Iron Age)	51	—	−22.2 ± 0.3	6.3 ± 0.7	20	—	−22.6 ± 0.4	6.1 ± 0.9	0.33	**0.001**	0.16	0.428
Mountgorry (early medieval)	9	—	−22.0 ± 0.2	8.0 ± 1.2	16	—	−22.2 ± 0.3	6.5 ± 0.9	0.27	**0.022**	1.50	**0.002**
Navan Road (early medieval)	6	—	−21.8 ± 0.3	9.1 ± 1.0	18	—	−22.1 ± 0.4	7.4 ± 0.7	0.29	0.101	1.70	**<0.001**
Ratoath (early medieval)	12	—	−21.9 ± 0.4	9.3 ± 1.2	15	—	−22.3 ± 0.4	7.9 ± 1.0	0.39	**0.025**	1.34	**0.003**
Stalleen (early medieval)	12	—	−21.8 ± 0.3	7.9 ± 0.6	15	—	−21.9 ± 0.2	6.4 ± 0.4	0.04	0.660	1.53	**<0.001**
Greencastle (later medieval)	5	—	−21.5 ± 0.3	7.8 ± 1.1	23	—	−22.5 ± 0.4	5.6 ± 0.8	0.99	**<0.001**	2.18	**<0.001**
Nobber (later medieval)	17	—	−21.5 ± 0.2	7.7 ± 0.8	9	—	−22.6 ± 0.6	6.1 ± 1.3	1.10	**0.001**	1.55	**0.001**
Eyre Square (post-medieval)	6	—	−21.8 ± 0.5	8.0 ± 1.4	11	—	−22.2 ± 0.4	6.2 ± 1.2	0.40	**0.024**	1.84	**0.010**

The Neolithic–Early Bronze Age sample includes isotopic compositions from two specimens identified by the original zooarchaeologists as wild boar [43,50]. We note that these samples fall within the main clustering of data (electronic supplementary material, figure S2), and their exclusion does not change Neolithic–Early Bronze Age sample means significantly (table 2). Due to the small size of our sample from this early period, we include data from these specimens in the Neolithic–Early Bronze Age group in the following comparisons.

During sampling, bones were identified and recorded by an experienced zooarchaeologist. Due to specimen fragmentation, which prevented direct observation of age-related morphological indicators, it was not always possible to establish age based on fusion; however, care was taken to exclude samples from small and therefore young juvenile pigs. Given the relatively short window of milk suckling for piglets, which naturally wean by 17 weeks and may be artificially weaned before this [51], and the efforts we have made to exclude samples from this age category, we do not expect that patterns in pig isotopic variation are driven by this factor. Two pig specimens, both from historical contexts at the coastal site of Greencastle, County Down [52], show isotopic compositions consistent with some consumption of marine foods. While these samples do not pass the conservative quality control criteria we use in this study [53], they would pass liberal quality control (see §6) and we offer brief interpretations in electronic supplementary material, text S1 and figure S3.

**Figure 3.**
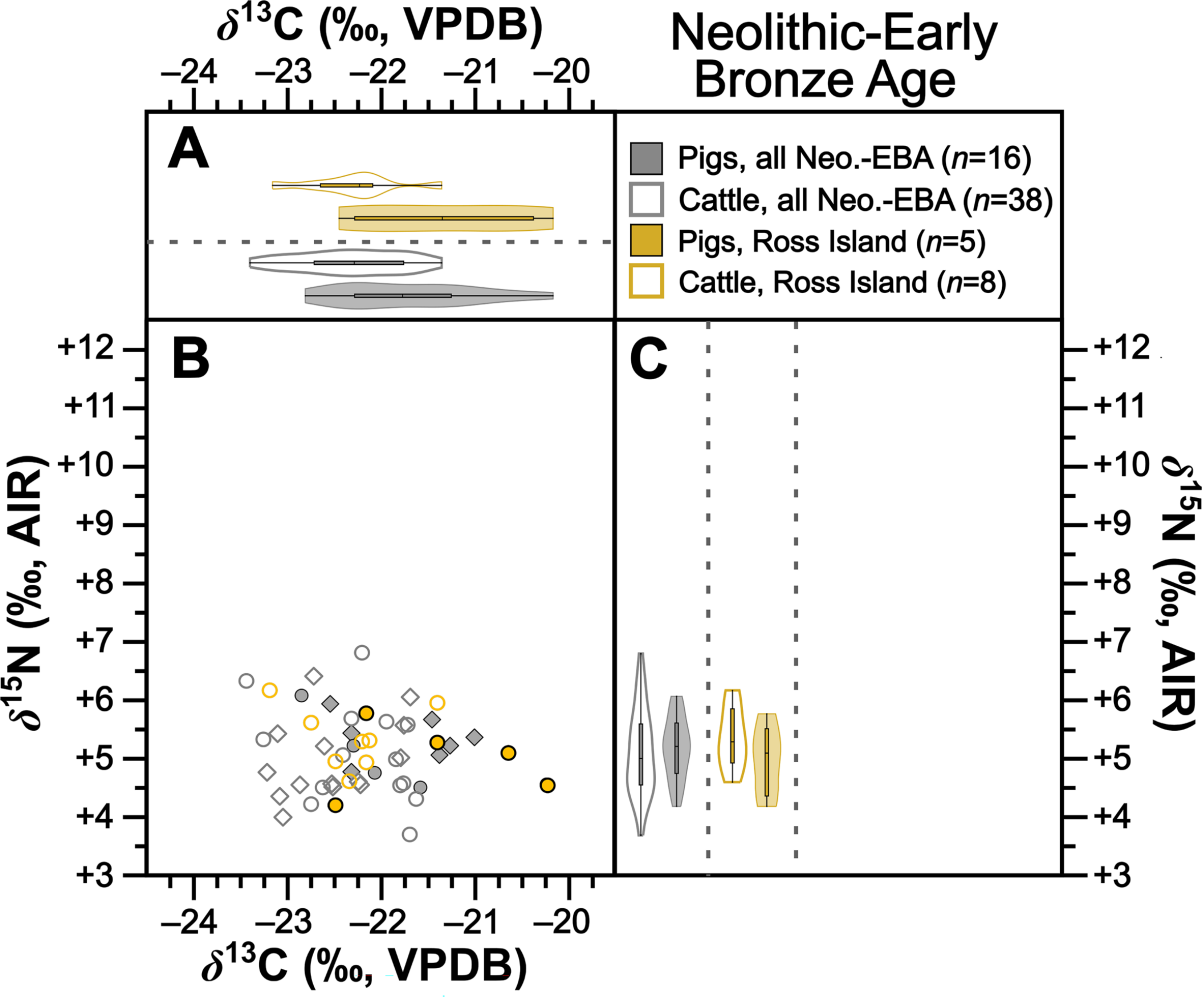
Isotopic compositions of Neolithic and Early Bronze Age pigs and cattle shown in bivariate space (*b*) and as violin and box plots showing kernel density for *δ*^13^C (*a*) and *δ*^15^N (*c*). Data are shown by site for sites with isotopic compositions for at least five individuals from each taxon. For (*b*), symbols are grey for sites with fewer than five individuals from each taxon. For statistical significances, see table 2.

**Figure 4.**
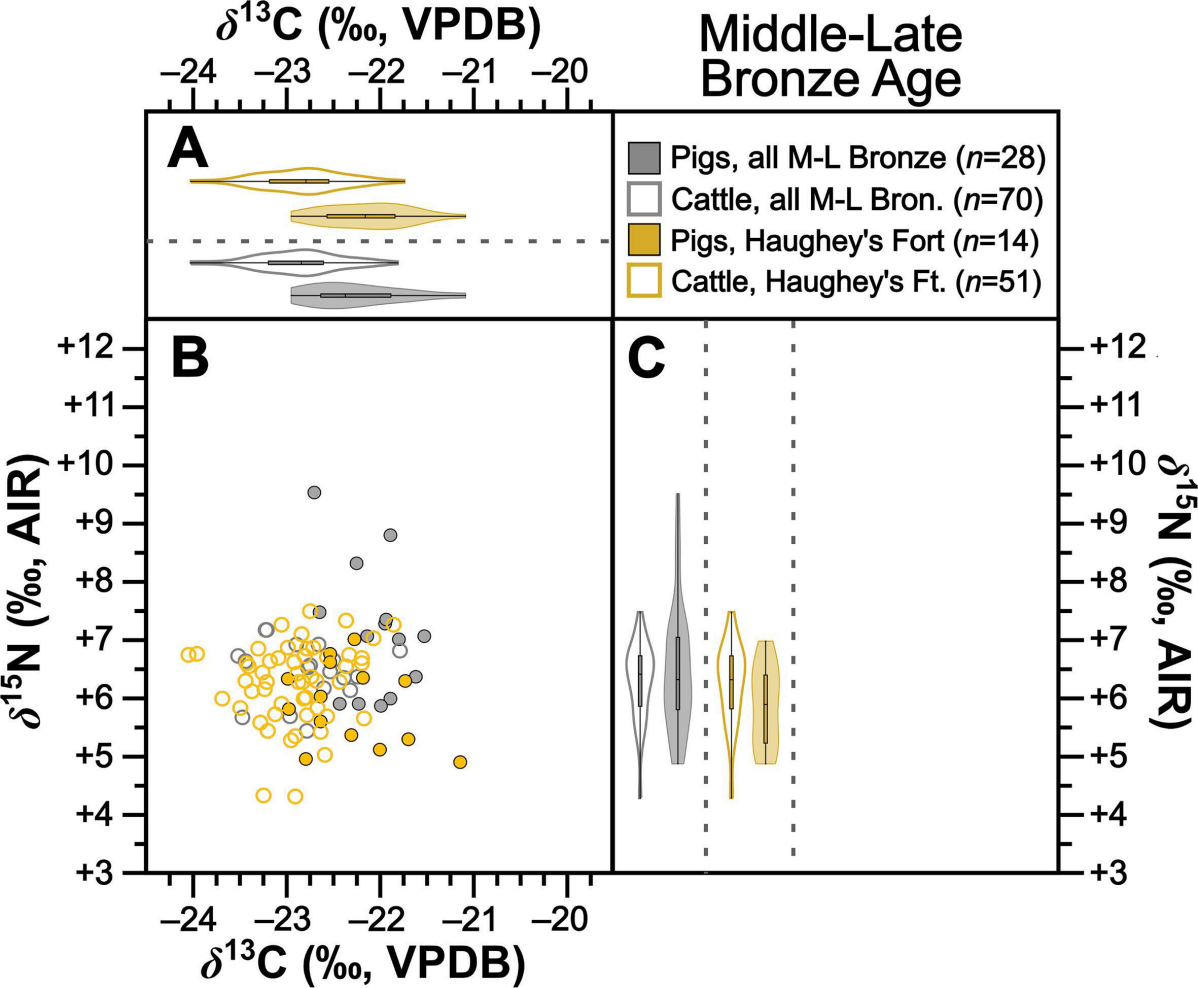
Isotopic compositions of Middle–Late Bronze Age pigs and cattle shown in bivariate space (*b*) and as violin and box plots showing kernel density for *δ*^13^C (*a*) and *δ*^15^N (*c*). Data are shown by site for sites with isotopic compositions for at least five individuals from each taxon. For (*b*), symbols are grey for sites with fewer than five individuals from each taxon. For statistical significances, see table 2.

**Figure 5.**
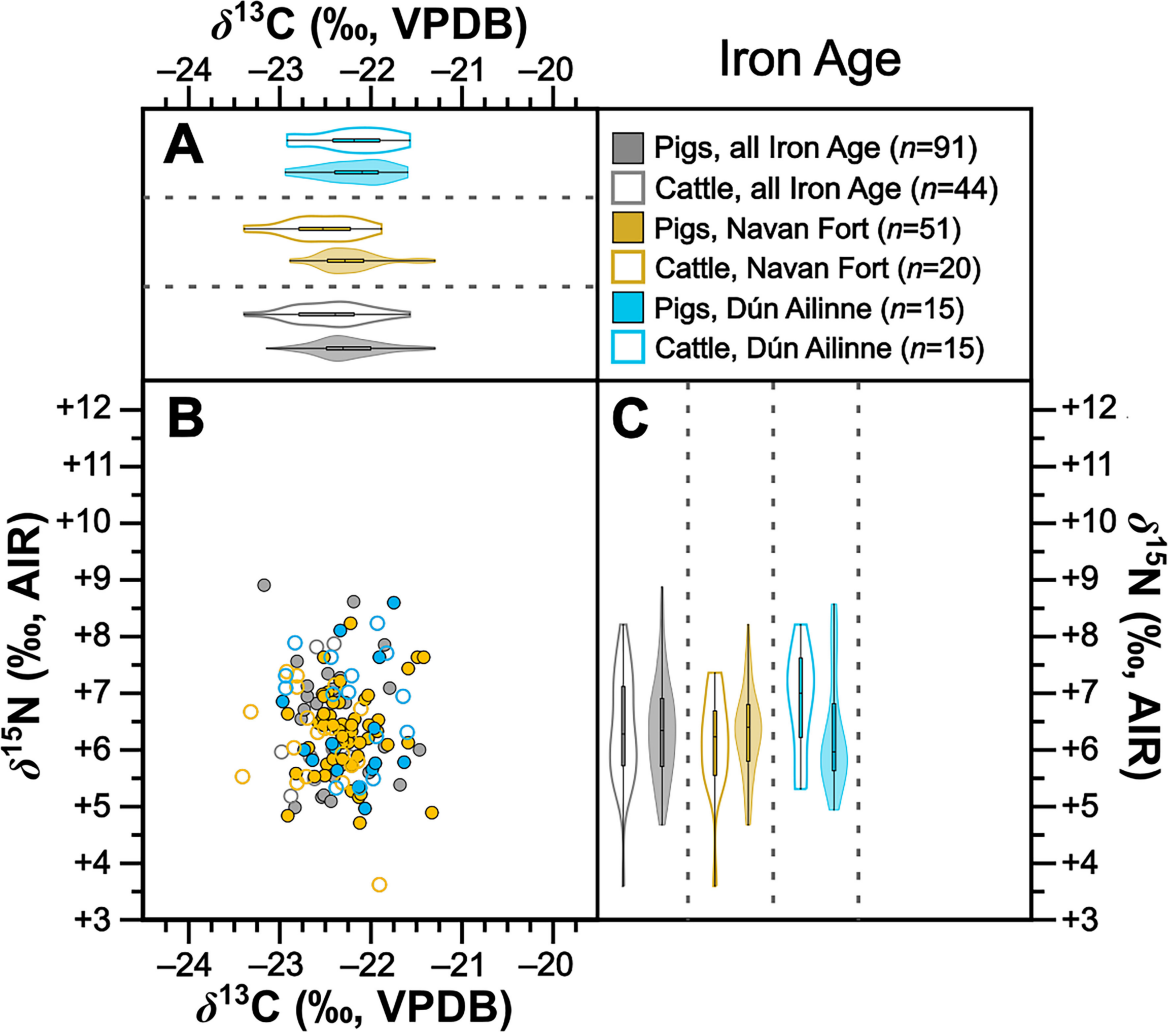
Isotopic compositions of Iron Age pigs and cattle shown in bivariate space (*b*) and as violin and box plots showing kernel density for *δ*^13^C (*a*) and *δ*^15^N (*c*). Data are shown by site for sites with isotopic compositions for at least five individuals from each taxon. For (*b*), symbols are grey for sites with fewer than five individuals from each taxon. For statistical significances, see table 2.

**Figure 6.**
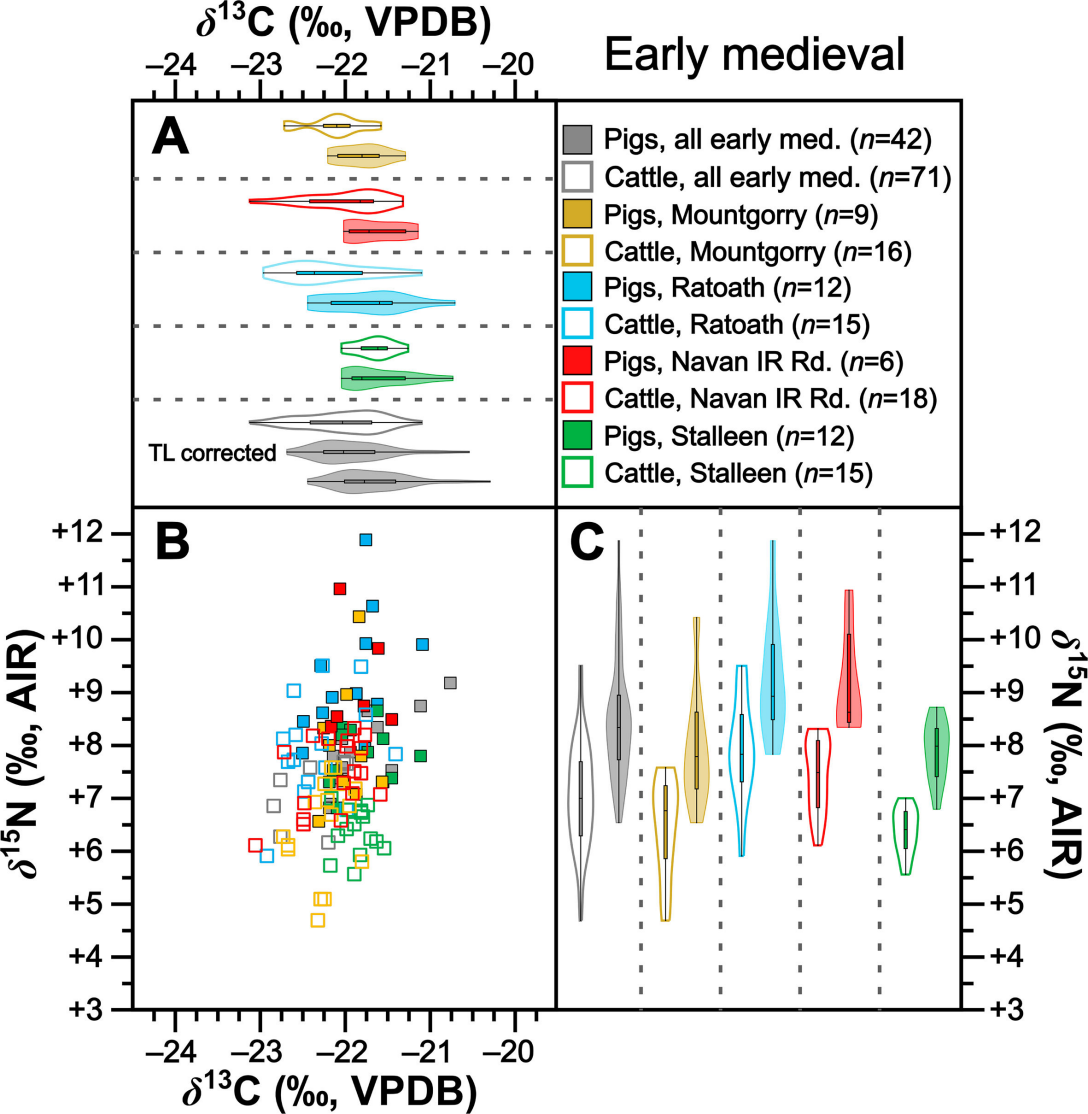
Isotopic compositions of early medieval pigs and cattle shown in bivariate space (*b*) and as violin and box plots showing kernel density for *δ*^13^C (*a*) and *δ*^15^N (*c*). Data are shown by site for sites with isotopic compositions for at least five individuals from each taxon. For (*b*), symbols are grey for sites with fewer than five individuals from each taxon. For (*a*), a second violin plot for early medieval pigs grouped by time period (‘TL corrected’) shows trophic-level adjusted *δ*^13^C (per electronic supplementary material, table S10). For statistical significances, see table 2.

**Figure 7.**
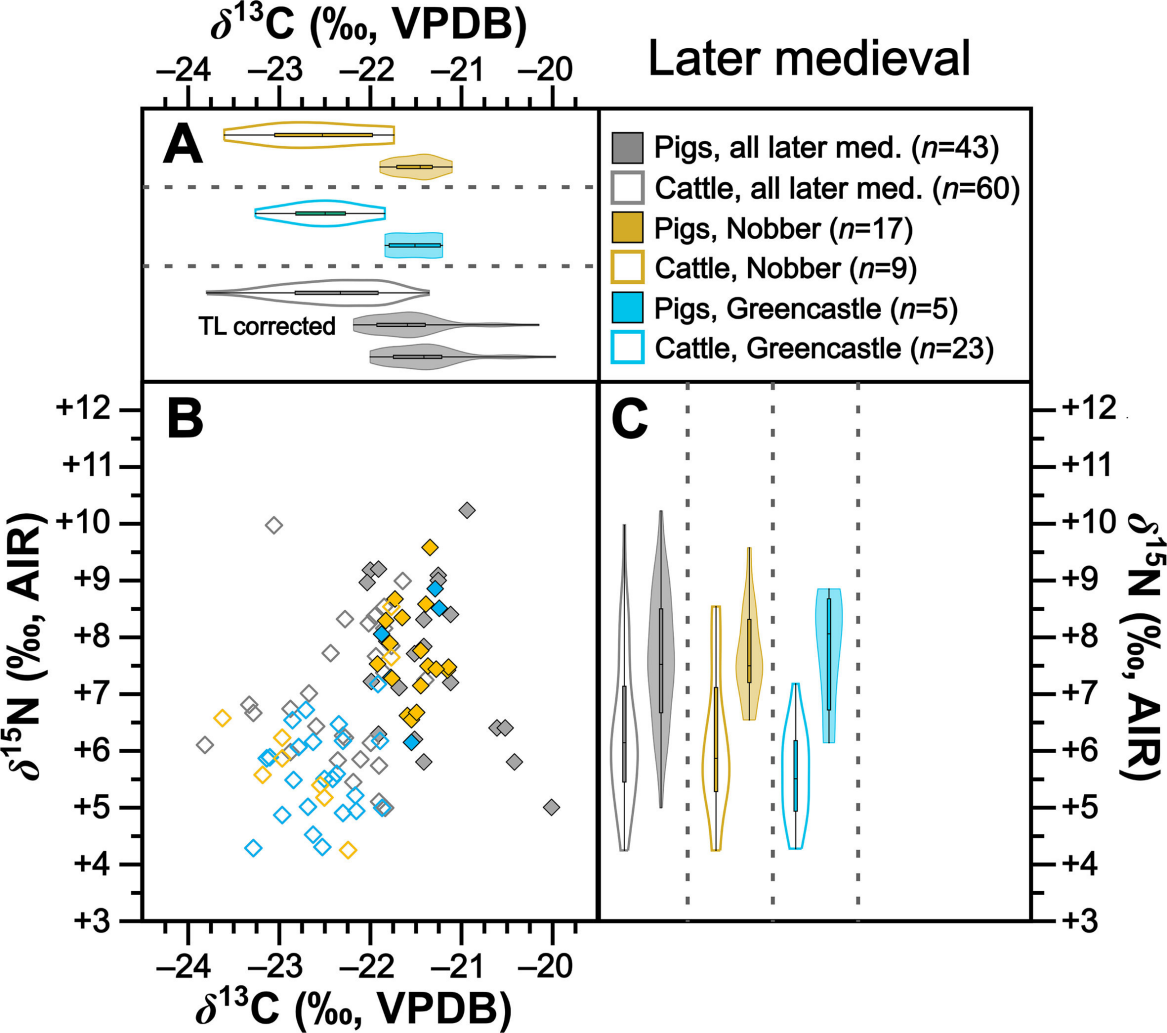
Isotopic compositions of later medieval pigs and cattle shown in bivariate space (*b*) and as violin and box plots showing kernel density for *δ*^13^C (*a*) and *δ*^15^N (*c*). Data are shown by site for sites with isotopic compositions for at least five individuals from each taxon. For (*b*), symbols are grey for sites with fewer than five individuals from each taxon. For (*a*), a second violin plot for later medieval pigs grouped by time period (‘TL corrected’) shows trophic-level adjusted *δ*^13^C (per electronic supplementary material, table S10). For statistical significances, see table 2.

**Figure 8.**
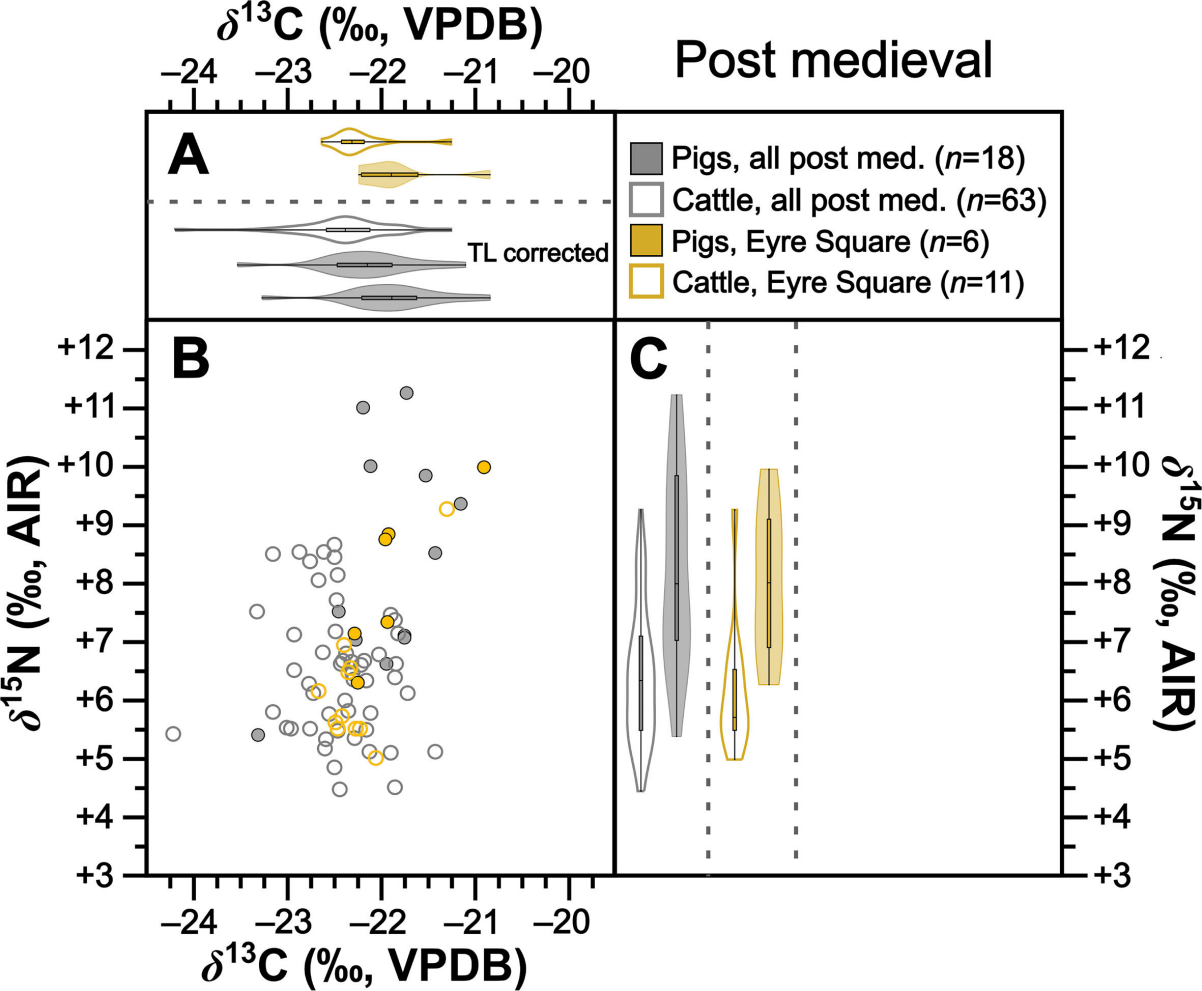
Isotopic compositions of post-medieval pigs and cattle shown in bivariate space (*b*) and as violin and box plots showing kernel density for *δ*^13^C (*a*) and *δ*^15^N (*c*). Data are shown by site for sites with isotopic compositions for at least five individuals from each taxon. For (*b)*, symbols are grey for sites with fewer than five individuals from each taxon. For (*a*), a second violin plot for post-medieval pigs grouped by time period (‘TL corrected’) shows trophic-level adjusted *δ*^13^C (per electronic supplementary material, table S10). For statistical significances, see table 2.

Collectively pigs show considerable *δ*^13^C and *δ*^15^N variation (figure 2). To establish how much of this variation is driven by baseline versus dietary change, we compared mean pig with cattle isotopic compositions grouped by time period (table 2). During the Neolithic–Early Bronze Age (figure 3*b*,*c*), Middle–Late Bronze Age (figure 4*b*,*c*) and Iron Age (figure 5*b*,*c*), mean pig *δ*^15^N values are close to those of cattle from their respective time periods (means differ by 0.0–0.2‰), with no significant differences (table 2). This suggests that during these prehistoric periods, pigs were largely herbivorous. By contrast, during the early (figure 6*b*,*c*), later (figure 7*b*,*c*) and post-medieval (figure 8*b*,*c*) periods, mean pig *δ*^15^N was substantially higher than that of cattle (means differ by 1.3–1.8‰) and consistently shows significant differences (table 2). This suggests that during the historic period, pigs were largely omnivorous (see §4). With respect to *δ*^13^C, across all temporal comparisons (figures 3–8, panels (*a*) and (*b*)), mean pig *δ*^13^C values were consistently elevated (by 0.2–0.9‰) above, and were in all cases significantly different from, cattle from their respective time periods (table 2). These interspecific *δ*^13^C differences indicate that, relative to cattle, pig diets were on average focused on more ^13^C-rich foods (for consideration of interspecific differences in digestive physiology, see §6).

To further verify these interspecific time-period-level patterns, we also perform the same comparisons between means for isotopic compositions of cattle and pigs at the same sites, using data from all sites with at least five cattle and five pigs from one time period (table 2). Such comparisons could be made at one Neolithic–Early Bronze Age, one Middle–Late Bronze Age, two Iron Age, four early medieval, two later medieval and one post-medieval site (figures 3–8 panels (*b*) and (*c*)). Of these 22 comparisons, nearly all (11 of 11 for *δ*^15^N and 8 of 11 for *δ*^13^C) conform to the patterns observed at the time-period level in terms of both order of means (e.g. where pigs had higher means than cattle) and statistical significance (table 2). With respect to the three exceptions (Dún Ailinne, Ratoath, Navan Inner Relief Road 1–3), in all instances pigs still produced mean *δ*^13^C values that were higher than their respective cattle group, but these differences were not statistically significant (table 2). These interspecific site-level comparisons, therefore, broadly confirm and support the picture we see at the time-period level. Moreover, the fact that these sites, located across several counties in Ireland (electronic supplementary material, figure S1), show isotopic patterns that correspond with those of their broader-scale temporal period, support our expectation, based on previous *δ*^15^N baseline work [43], that isotopic patterns are driven more by temporal than geographical factors. We can also note that these sites include a range of socio-economic situations, including sites that are more rural and more urban, and sites of higher and lower status (see electronic supplementary material, table S1), suggesting that patterns transcend these site type categories (see §4).

For the Iron Age, there is a particularly large sample of pigs, which is >50% composed of specimens from a single site, Navan Fort. To establish that temporal patterns in pig–cattle comparisons for this period were not driven by relatively localized phenomena influencing pig diets at Navan Fort, we also compare pigs from Navan Fort (*n* = 51, mean *δ*^13^C = −22.2 ± 0.3‰, Shapiro–Wilk *W* = 0.945, *p* = 0.020; mean *δ*^15^*n *= +6.3 ± 0.7‰, Shapiro–Wilk *W* = 0.984, *p* = 0.707) to the rest of the sample of pigs from the Iron Age period, which are sufficiently similar to be pooled (*n* = 40 from five sites, mean *δ*^13^C = −22.3 ± 0.4‰, Shapiro–Wilk *W* = 0.983, *p* = 0.798; mean *δ*^15^*n *= +6.5 ± 1.1‰, Shapiro–Wilk *W* = 0.947, *p* = 0.060). No significant differences were observed for either *δ*^13^C (Mann–Whitney *U* = 954, *p* = 0.600; Vargha–Delaney *A* = 0.532) or *δ*^15^N (Mann–Whitney *U* = 957, *p* = 0.885; Vargha–Delaney *A* = 0.482). This comparison suggests that the relatively large number of samples from Navan Fort has not disproportionately influenced Iron Age isotopic patterns.

To evaluate the significance of chronological change in levels of pig omnivory across time we also compared baseline-corrected mean *δ*^15^N between time periods (compare figure 2*b*,*c*). This was accomplished by sequentially comparing mean pig *δ*^15^N between adjacent time periods but, for each comparison, we adjusted the latter temporal groups’ *δ*^15^N values according to the baseline offset observed between groups of cattle from those respective time periods (electronic supplementary material, tables S8 and S9; see §6 for more detail). Differences in baseline-corrected mean pig *δ*^15^N between time periods were all small (within 0.5‰) except for during the Iron Age to early medieval transition, which showed an upward *δ*^15^N shift of 1.4‰ after baseline adjustment (figure 2*c*; electronic supplementary material, table S9). Statistical significances for these comparisons follow the same pattern, with no significant differences (i.e. *p* ≤ 0.05) observed except for the comparison between the Iron Age and the early medieval groups (where *p* ≤ 0.001; electronic supplementary material, table S9). Together these comparisons support the trends observed in cattle–pig comparisons between time periods. Specifically, pig *δ*^15^N consistently shows a herbivorous diet from the Neolithic until the end of the Iron Age. Pig *δ*^15^N then underwent an upward shift reflecting a substantial increase in intake of animal protein in the early medieval period, after which time this meaningful level of omnivory remained relatively consistent until the final, post-medieval timeframe.

Lastly, given the complexities of discerning the relative impact of the two major sources of *δ*^13^C variation (i.e. the canopy effect versus mast/grain consumption; see table 1 and §4), we compare *δ*^13^C means from pigs grouped by time period to cattle from the early medieval period [23]. The high *δ*^13^C observed in cattle during the early medieval is thought to reflect the zenith for open-land pasturing (for consideration of climatic factors on *δ*^13^C, see §6), and provides a convenient benchmark beyond which mast consumption is more likely. In nearly all cases, pig *δ*^13^C means were higher than those of early medieval cattle. The exception being Iron Age pigs, with a mean *δ*^13^C that is 0.1‰ lower than that of cattle (see electronic supplementary material, table S10, for results). Differences were significant for comparisons involving Neolithic–Early Bronze Age, later medieval, and post-medieval pigs, but not for the Middle–Late Bronze Age and Iron Age comparisons (electronic supplementary material, table S10).

Considering the higher-trophic-level nature of pigs from the early, later and post-medieval periods, we compared the *δ*^13^C means of these groups, adjusted for trophic ^13^C enrichment, to cattle from the early medieval period (electronic supplementary material, table S10; see §6). Pig trophic level (TL_pig_) for the early, later, and post-medieval periods ranged from 0.4 to 0.5 (electronic supplementary material, table S10; see §6). In all cases, mean pig *δ*^13^C was higher than that of early medieval cattle (by 0.2–0.3‰), but the difference was only significant for the later medieval comparison (electronic supplementary material, table S10).

## 4. Discussion

Results from 238 specimens show considerable variation in pig isotopic compositions across the later Irish Holocene (*ca* 4000 BC to *ca* AD 1900; see figures 2c,6,7 and 6–8*a* for baseline-corrected data). Considering pig isotopic compositions relative to published cattle data, which serve as a faunal baseline (see §6), two patterns emerge. On the one hand, a striking and comparatively rapid change in pig trophic behaviour occurs at the transition from the Iron Age to the early medieval period. On the other hand, there appears to be 6000 years of continuity in use of mast from woodlands, the same resource that we know from historical records [12] had become synonymous with pig husbandry by the early medieval period. We will consider the isotopic evidence for each of these processes, and their attendant implications, in turn.

### 4.1. Pigs and proximity: nitrogen isotope compositions show change

One starting point for considering change in pig diets is the diet of wild boar. As noted above, these are naturally woodland dwelling and generally herbivorous, subsisting on mast in the autumn/winter and leaves, seeds, fruit and roots throughout the year. Limited animal protein in their diet may include carrion, young mammals and birds, and eggs [5]. This ecological niche is evidenced by two zooarchaeologically identified wild boar samples (electronic supplementary material, figure S2) showing the expected predominantly herbivorous diets. These results are isotopically similar to those of zooarchaeologically identified domestic pigs at that time. This similarity suggests that Neolithic and Early Bronze Age pig management may have allowed pigs to mimic their wild counterparts.

This approach to pig management continued for millennia. Binned by time period and corrected for baseline changes, mean *δ*^15^N values (figure 2*c*) clearly show that during the Neolithic–Early Bronze Age, Middle–Late Bronze Age and Iron Age, pigs were largely herbivorous. By contrast, pigs raised during the early, later and post-medieval periods were omnivores with considerable animal-protein intake (potentially including both meat and dairy; ranging from TL 0.4 to 0.5, where 0 is fully herbivorous, and 1 is fully carnivorous; electronic supplementary material, table S10).

Before exploring broader *δ*^15^N trends as evidence for changing Irish pig husbandry practices, it is worth pointing out that, at the individual specimen level, there are likely some exceptions to the overall pattern of prehistoric pig herbivory, particularly during the Middle–Late Bronze and Iron Ages. Previous work has documented *δ*^15^N baseline shifts, which began to move dramatically sometime in the Middle–Late Bronze Age and onwards [43]. In aggregate, at the island-wide scale, these baseline changes appear to occur in unison. However, we expect that if sufficient data were to become available to examine how these changes unfolded over smaller segments of space and time we would see a heterogeneous patchwork of isotopic compositions with changes happening at different rates in different places centring on the later Bronze Age. In that context, moving from the aggregate time-period level to assess diet at the individual level, it becomes difficult to evaluate the extent to which isotopic compositions from specific pigs represent higher trophic positions (i.e. more omnivorous diets) rather than fluctuations in ^15^N content of local plants and other food sources, such as those which can arise from changing cultivation and other land management processes [40]. However, considering the particularly high *δ*^15^N values for some specimens from the Middle–Late Bronze Age (e.g. samples with *δ*^15^N > *ca* 8.0‰, *n* = 3 of 28, electronic supplementary material, table S6, figure 4*b*, some of which were definitely adults) it seems likely that at least some pigs were consuming diets rich in animal protein at this time. Nonetheless, at scale, and compared with the early medieval, the evidence we have generated for later prehistoric pigs still suggests that this was a comparatively rare husbandry pathway at that time. In that context, it is worth noting that a very small (+0.2‰) but significant elevation was observed in mean *δ*^15^N of pigs relative to cattle during the Middle–Late Bronze Age (table 2) suggesting that pigs in this time period could have consumed slightly more animal protein on average than would be expected for herbivores. However, this ^15^N enrichment is small enough (representing only *ca* 0.05 TL, or about 5% of a trophic level step from herbivore to carnivore) that it remains inconsequential for our interpretations. We further note that during the Iron Age, pigs and cattle produced virtually identical mean *δ*^15^N values (table 2). On the whole, it is therefore clear that, like their Neolithic and Early Bronze Age counterparts, later prehistoric pig keepers did not take pains to involve substantial amounts of animal protein in pig diets.

To explore the temporal shift observed in mean pig *δ*^15^N (figure 2*c*, table 2), we consider the implications from two perspectives. First, we reflect on what these patterns mean for our understanding of animal husbandry before the early medieval. Second, we ask what structural changes must have occurred at the early medieval juncture and subsequent periods to generate these patterns.

In order to unpack what these isotopic patterns mean for our understanding of prehistoric pig diets, it is best to start in the middle of the story, and ask why it was that pigs would have begun consuming large quantities of animal protein and what processes drove that change. It is widely held that pigs will eat virtually any food, but among this almost limitless potential menu, sources of animal protein are particularly valuable from the perspective of both the pigs and their associated keepers. We know, for instance, that, granted access, pigs will consume meat and other animal products preferentially [54,55]. Because pigs will readily consume animal products, and feeding animal-protein-rich food to pigs helps increase weight gain [55,56], it is likely that knowledge of the value of adding animal protein from waste products of human activity to pigs’ diets is of nearly the same antiquity as pig husbandry itself.

In that context, if we accept as our baseline premise that feeding edible animal-protein-rich waste from human activities to pigs has always represented a potentially lucrative pathway to generating more pork, it is reasonable to ask why pigs husbanded in Ireland prior to the early medieval evidently consumed so little animal protein. Taking a hypothetical approach, one could hypothesize that there was less human-generated animal protein to go around and, therefore, that on average very little animal protein could be diverted from human, or indeed other domesticate (i.e. dog), use towards raising pigs. In turn, this would suggest that sources of human-generated animal protein (i.e. livestock agriculture and hunting and fishing activities) were less productive during prehistory. Against the wider corpus of evidence from prehistoric agricultural practice [57], however, it seems implausible to suggest that, on average, areas of the landscape with denser prehistoric human populations (e.g. settlements, farmsteads) would not have generated meaningful amounts of edible animal-protein-rich waste. In that context, an alternative approach is to ask why prehistoric people would apparently ignore this special, and arguably one of the most economically valuable, dimensions of pig husbandry—their capacity to convert human-generated sources of waste, especially those that are rich in animal protein, into more food for humans [56].

We note that cultural factors could offer one possible explanation. For instance, it is conceivable that the consumption of animals may have been seen as culturally inappropriate for pigs, perhaps because this behaviour would make pigs too close to humans. These possibilities, however, remain speculative and, while the limited nature of existing evidence for prehistoric human–pig relationships in Ireland [13] means that this potential issue cannot be adequately assessed, we assume that it does not offer a more parsimonious explanation than scenarios in which pigs living near humans could access a representative selection of human-generated food scraps.

With the backdrop of these questions and the apparent interpretive paradox to which they give rise (i.e. why prehistoric people did not direct available edible animal-protein waste to raising pigs), we can offer a more parsimonious explanation—that in the context of prevailing prehistoric settlement patterns, environmental resources and pig ecology, it made less sense to prehistoric farmers to husband pigs in ways that involved comparatively animal-protein-rich foods. More specifically, our data may reflect a scenario in which prehistoric pig husbandry systems in Ireland were more often centred (for most of the year anyhow) away from areas with denser human populations. For our purposes, areas of denser human population would be settlement locations associated with a sustained availability of human-generated animal protein (i.e. food scraps, agricultural byproducts, human excrement), such as small concentrations of farms or even individual farmsteads. Reasons for this may have been manifold, involving rationales embedded in seamless human–animal–environmental worldviews that, today, we might recognize as being not only economic, but social, cultural and religious in nature. While we have little concrete evidence with which to explore the latter, we can consider some of these processes in terms of the opportunities they offered farmers to generate larger numbers of pigs and thus more pork.

In that context, we can hypothesize both ‘pull’ and ‘push’ factors that could have served to guide pig husbandry locations away from where more people lived. We recognize that describing ‘pull’ factors in this way presupposes that pig husbandry has a default setting in which pigs will be husbanded close to people. Indeed, as our data suggest, this may not have been the case in the distant past and, instead, the ‘default’ approach to early pig husbandry could be management of pigs in locations away from denser human areas. After all, these woodlands were the same habitats preferred by wild boars, making them ecologically well suited for domestic pigs as well. Acknowledging this issue of presentism, we proceed using these terms to frame our interpretation as the ‘push–pull’ spectrum serves to neatly illustrate potential trends.

With respect to factors that could have pulled pig husbandry away from sources of human-generated waste, it may have been that woodlands and other ecosystems further afield were considered more productive habitats for raising pigs than locations such as settlements or other areas with relatively dense human populations that were richer in human-generated animal protein. In a scenario where highly productive ecosystems for pigs were abundant and widely accessible, communal husbandry (via swine herders) could have offered a better approach to pork production than more blended systems that were traditional by the early medieval period (e.g. involving rearing of pigs at individual farmsteads, ringforts, for a large portion of the year, followed by communal swine herding during the masting season; see below). An alternative, though not mutually exclusive factor could be that prevailing settlement patterns were less conducive to keeping pigs on farms or in settlements during the prehistoric period. We know comparatively little about settlement patterns in Ireland in later prehistory (for a review, see [58]), but it is conceivable that they were organized in such a way that keeping pigs closer to human habitation would have represented a liability (relative to later periods) and that this served as a ‘push’ factor. Such liabilities could stem from differences in defensibility, mobility or care and management needs of pigs relative to other more important foci of activities in areas with denser human groups (i.e. the places we would expect humans to generate more animal-protein-rich waste for potential pig feed).

While the prehistoric pig isotopic data alone cannot serve as a basis for weighing the relative influence of the potential ‘pull’ (of other habitats) or ‘push’ (of potential incongruencies of farm-based pig husbandry and the other responsibilities faced by prehistoric farmers) factors, additional perspective can be gained from taking a longer view. Zooming out to consider the dataset as a whole, what is abundantly clear is that whichever factor drove the animal-protein-light diets of prehistoric pig husbandry, the ‘barriers’ to connecting pigs with human-generated animal-protein-rich food waste had disappeared or been overcome by the early medieval period. Or, put in less presentist terms, farmers, for one reason or another, chose to husband pigs in a different way that included more animal protein. The emergence of these new animal husbandry practices with comparatively animal-protein-rich diets during the early medieval provides a historically referenced anchor point from which to retrospectively reconsider these potential ‘push’ and ‘pull’ factors in prehistory.

Textual evidence for early medieval Irish agriculture provides a robust framework for fleshing out the kinds of husbandry practices responsible for generating what, in the context of our dataset, represented a blending of new and traditional (see below) forms of animal husbandry. As in the prehistoric period, during the medieval and post-medieval periods, the richest sources of animal protein for pigs would have been areas of concentrated human activity. Specifically, pigs were raised in connection with a broad range of ready sources of human-generated animal-protein-based foods, including anything along a spectrum from household-level waste to byproducts, such as whey, from farmstead-level to quasi-industrial-scale monastic operations [12,20]. What is most salient for considering patterns in prehistoric husbandry are sources showing that, on average, medieval pig husbandry also involved a substantial focus on pannage in woodlands [12] (see also [59,60]). In turn, this suggests that woodland resources, situated at some remove from human settlements, were still being used for pig husbandry during part of the year. In that context it is fair to ask, if typical approaches to early medieval pig husbandry still involved the travel and time spent away from human population centres for pannage (as evidenced in both historic and prehistoric pig *δ*^13^C values; see below), what other changes account for the contrast of pigs spending the remainder of their time in closer proximity to more densely populated areas (as evidenced by greater access to animal protein during the historic period)?

The thirteenth-century agriculturist and widely read English scholar Walter of Henley noted that it was much more economical to rear pigs ‘in the forest, or in woods, or waste, or in marshes’ rather than at the farm [61]. He continues that:

for whoever will keep swine for a year from the cost of the grange [farm] alone, and count the cost and the allowance for the swine and swineherd, together with the damage they do yearly to the corn, he shall lose twice as much as he shall gain, and this will soon be seen by whoever keeps account.

It may therefore be suggested that the change in strategy to farm-based rearing of pigs in early medieval Ireland was occasioned by necessity rather than choice. Several broad factors could explain this.

Plunkett [62] notes that there was extensive woodland clearance throughout much of Ireland during the early medieval period, which, in turn, could have restricted the availability of mast. The documentary evidence also suggests that woodland was not as extensive and tended to be confined to poorer, marginal land [12], the implication being that woodland on better lands tended to be cleared for agricultural use. However, there is no evidence for sudden widespread woodland clearance with the onset of the early medieval. It was a gradual process occurring at different times in different parts of the country [63]. This decline in woodland alone cannot account for the sudden change in pig diet at the beginning of the period. Moreover, at least some woodland survived, providing opportunities for pannage in at least some regions in later periods.

The second possible factor is changes in land ownership and settlement. The documentary evidence indicates that much of the surviving woodland was privately owned [12], thus potentially restricting pannage rights and mast availability for many, if not most, farmers. The early laws indicate ‘that early Irish society attached great importance to the principle of the private ownership of property’ [64]. Indeed Mytum [65] argued that the change from kin-group land ownership, which would have facilitated more general pannage rights, to private land ownership coincided with the advent of Christianity in Ireland. A sudden change in settlement also occurs at this time. The settlement type in Iron Age Ireland was of dispersed rural unenclosed round houses, which was essentially unchanged since the Bronze Age [66]. The roundhouse tradition continued but in the early medieval period the house became surrounded by a formidable ditch. The enclosed, defended, privately owned farmsteads (i.e. ringforts), which number in their tens of thousands, now became the type-site. The law tracts imply that pig sties and pens for other domesticates were located within the ringforts [12], and the presence of pigs is demonstrated by an abundance of pig lice (specifically *Haematopinus apri*) recovered from a waterlogged ringfort level at Deer Park Farms, County Antrim [67]. Perhaps a change to private ownership of land and livestock somehow necessitated the change in strategy of rearing pigs from a free-range regime of woodland and wasteland to one where, for the majority of the year, they were reared at the farm, with short periods of pannage in woodland, if available. Indeed, it has been argued that the ringfort was devised primarily to protect livestock rather than humans [68].

A third factor to be taken into consideration is the increasing importance of cattle in the early medieval period [23,68]. This would have led to increased availability of milk and associated byproducts such as whey and buttermilk. These byproducts of the cheese- and butter-making processes represent potentially valuable sources of animal protein for pig husbandry. While some would have been used for human consumption, they have been important elements in pig feed up to modern times. Their availability, therefore, could represent an important ‘pull’ factor, drawing pig husbandry closer to areas of human habitation.

Having considered processes that may have contributed to husbandry of pigs in closer proximity to humans in the medieval and post-medieval periods, we can now return to our consideration of ‘push’ and ‘pull’ factors responsible for more distant pig husbandry practices in the prehistoric period. While there remain questions of the relative scale of different potential pools of pig food resources, the overall pattern seems to suggest that something more fundamental or structural shifted approaches to settlement between the prehistoric and early medieval periods, which made husbandry of pigs in or near human habitations more preferable. In other words, the ‘push’ factors had diminished and the pull factors (e.g. availability of dairy byproducts; ringforts for livestock defensibility) had increased. In that context, it could be that changing social, political, and economic forces during the transition from late prehistory to the early medieval period altered the landscape of risks and benefits associated with husbandry of pigs in areas further from farmsteads and other centres of agricultural activity. Whatever the cause, when considered in the light of the early medieval texts, which demonstrate that husbandry in areas away from settlements continued on a seasonal basis [12], the trends we see in the prehistoric period suggest that shifting economics and settlement patterns could have been drivers of the changing approaches to husbandry evidenced in pig *δ*^15^N.

### 4.2. Of mast and meat: carbon isotope compositions show consistency

In contrast to the *δ*^15^N evidence, a dietary trend that is consistent across the dataset is that pig *δ*^13^C values are broadly in line with at least some pannage feeding throughout all time periods. We recognize that opportunities for pannage husbandry would have been diminished as woodlands were converted to open fields in the medieval and post-medieval periods, but, as outlined in early medieval texts, pannage continued where surviving woodlands allowed [12,69,70]. Pigs show significantly higher mean *δ*^13^C relative to cattle in all respective time periods (figures 3–8; table 2). Pig *δ*^13^C means are also higher than those for respective cattle in all intra-site comparisons, most of which were statistically significant (figures 3–8; table 2). The main levers governing carbon isotope variation between cattle and pigs differ in ways that make their *δ*^13^C values challenging to compare directly (see above). Moreover, small systematic interspecific differences in how digestive physiology influences bone collagen isotopic compositions cannot be ruled out, though it is possible that these could serve to dampen, rather than artificially inflate, the interspecific pattern observed here (see §6) [71]. A result of these challenges is that patterns definitively identifying pannage will be rare when pig isotopic compositions do not sit near the extreme positive end of the *δ*^13^C range [72]. Cattle diets are, from an isotopic perspective, constrained relative to pigs. Because cattle are herbivores and cannot consume large quantities of mast [73,74], at this aggregated and island-wide scale variation in their isotopic compositions is primarily driven by one factor—the canopy effect—making interpretation of variation in cattle *δ*^13^C relatively straightforward [23]. By contrast, in addition to the canopy effect, *δ*^13^C variation in pigs will be influenced by two other ^13^C-enriching factors—animal protein (e.g. meat and dairy) and non-photosynthetic plant tissues (namely mast, but also grains). Each factor requires nuanced consideration before the question of pannage can be addressed.

First, to assess the impact of mast consumption on *δ*^13^C of pigs relative to cattle it is important to consider the carbon isotopic ecology of different food resources within woodlands and between woodlands and more open areas (table 1). This is because mast is produced in woodlands, a habitat in which typical foods will be ^13^C depleted relative to the same foods from more open areas [25]. For this reason, while mast is ^13^C enriched relative to woodland foliage [27,29], that ^13^C enrichment begins from a lower (i.e. more ^13^C-depleted) starting point. Mast consumption is thought to impart up to a 4‰ *δ*^13^C increase to the bone collagen isotopic compositions of mast consumers relative to foliage feeders in the same habitats [27,29]. If we consider the *δ*^13^C range across the entire published assemblage of archaeological cattle from all sites and time periods in Ireland as a proxy for extremes in woodland versus open-land pasturing, we see a spread of 2.9‰ (−24.2 to −21.3, *n* = 344, conservative C : N criteria applied) [23]. In that context, starting from the lower end of this observed *δ*^13^C range, even moderate mast consumption by pigs could result in *δ*^13^C values that fall within the range of cattle. It is also worth noting that this scenario represents a conservative interpretive framework because mast-generating woodlands do not always have dense canopies, and consumption of mast from more open woodlands would generate even higher (more distinctive) pig *δ*^13^C values [30].

Taking an even more conservative approach, rather than considering pig *δ*^13^C relative to cattle binned by respective time period, or even at individual sites, we can further compare pig *δ*^13^C means from all time periods relative to cattle from the early medieval period (electronic supplementary material, table S10). While anachronistic, this approach is more conservative because the early medieval is a period for which both archaeological and historical evidence suggest that open-land grazing (which should promote some of the highest possible terrestrial herbivore *δ*^13^C values outside of C_4_ consumption) of cattle reached its zenith [23,75]. Even in that more extreme comparative context we find that pigs still consistently have significantly higher mean *δ*^13^C values across time (electronic supplementary material, table S10). This comparison serves to underscore the extent to which pig *δ*^13^C is, by and large, higher than even the most ^13^C-enriched temporal grouping of non-mast feeders.

It is worth bearing in mind that, in addition to mast, husbandry could have provided pigs with diets rich in a wide range of other non-photosynthetic plant tissues. For instance, adding grains and cereals to pig feed should result in a ^13^C enrichment similar to mast consumption. In that context, particularly in the medieval and post-medieval periods, as brewing and distilling operations become more centralized and pigs more sty-based, grain-based waste could have joined mast as a factor elevating pig *δ*^13^C relative to cattle. While cattle would consume grain, they also consume these plants’ comparatively ^13^C-depleted photosynthetic tissues such as straw.

Second is meat and dairy consumption, which can result in a small TEF for *δ*^13^C (*ca* +0.5‰ per trophic level, though considerable variation has been noted [34]). As outlined above, consumption of animal protein has been established based on corresponding *δ*^15^N values, with pigs from the early medieval onward showing clear trophic differences from herbivorous cattle. It is therefore likely that medieval and post-medieval pig *δ*^13^C values are slightly elevated (by perhaps 0.2–0.5‰; corresponding to trophic levels of 0.4–0.5 based on mean cattle–pig *δ*^15^N offsets; electronic supplementary material, table S10) relative to herbivorous animals. This means that, in addition to pannage feeding, the larger differences we see between mean pig and cattle *δ*^13^C in early, later and post-medieval Ireland (table 2) may be amplified by a partial trophic offset. However, even factoring in these differences, mean pig *δ*^13^C from all three historical periods remain higher than the peak mean *δ*^13^C for early medieval cattle (though this difference is statistically significant only for the later medieval comparison; electronic supplementary material, table S10; figures 6–8). In other words, the *δ*^13^C differences we have observed between cattle and pigs are not attributable to trophic enrichment of ^13^C in pigs with diets richer in animal protein.

Having considered some of the nuances of the carbon isotope ecology of pig diets, the time series of *δ*^13^C we have presented are consistent with the expectation that mast would have been an important component of Irish pig diets. While variation in pig *δ*^13^C is observed through time, which may reflect an ebb and flow of the importance of mast and other non-photosynthetic plant tissues (e.g. grain), the complexities of carbon sources and cycling, compounded by variation from a ^13^C TEF, mean higher resolution interpretations remain out of reach without further analyses (see below). We also note that further work comparing our medieval and post-medieval results based on sites’ relative access to woodland and grain byproducts could allow for more detailed interpretations. For instance, pig *δ*^13^C variation between urban sites—which may have more access to grain byproducts and less access to mast—and castle and ecclesiastical sites—which would have better access to mast from private woodlands—could help unpack the cultural processes behind these patterns at a finer scale. Nonetheless, given that the centrality of pannage feeding in Irish pig husbandry is well documented in the earliest writing [12], and that woodlands are a natural habitat for wild boars, it is no surprise that our results suggest that this practice has been part of Ireland’s agricultural heritage since the beginning of farming on the island. This is supported by the similarity between the zooarchaeologically identified wild boars and contemporary domestic pig results presented in this study. In that context, it is worth pointing out that recent evidence has shown a degree of pannage-focused husbandry, including mean pig *δ*^13^C beyond what we have observed in this wider dataset, at Newgrange during the Late Neolithic (for discussion, see [72]).

### 4.3. Broader implications and consequences of changing pig proximity

We can also ask what these data mean for human–animal relationships in the medieval and post-medieval periods, eras that served as the precursors for present-day ontologies for understanding our own relationships to animals [76,77]. What implications, for instance, would husbandry of pigs closer to settlements and increases in the sharing of food resources have for humans? What other traces should these processes have left in the archaeological record? And what implications might the trajectory of changing approaches to pig husbandry in Ireland have for our understanding of pig husbandry in other regions of Europe?

Early medieval texts offer detailed information on the ways in which pigs were husbanded in Ireland, shedding considerable light on the roles that pigs played in early Irish societies. For instance, a rich tapestry of sources outline a wide range of norms about what pigs should and should not be fed, how many pigs should be kept and where they should reside relative to humans’ dwellings, and importantly when and for what duration pigs should be sent to woodlands [12]. These sources are invaluable for contextualizing our early medieval data. On the one hand, the sources explain, and reinforce many times over, the importance of animal protein, especially in the form of dairy products, in pig diets. On the other hand, the connection between pig husbandry and pannage in oak woodlands in the autumn is also a well-developed theme. We therefore have an interpretive framework that blends what had obviously been a long-standing tradition of woodland husbandry in the autumn/winter with new, more animal-protein-oriented feeding regimes during other times of the year. Together this set of practices seems tailormade to explain the isotopic compositions we have observed in early medieval pigs and highlights a new set of Irish pig–human relationships in which, compared with prehistory, the distance between where pigs were husbanded and where most humans lived was, on average, shorter.

Compared with the early medieval period, later and post-medieval pigs show similar dietary patterns, no doubt supported by a continuation of more closely quartered pigs in or near densely human-populated areas. However, in contrast to the early medieval period, when most settlement was of a dispersed, rural nature, these latter periods were a time of increasing urbanization, beginning with the arrival of the Vikings and then the Anglo-Normans [16,17,20]. The more frequent presence of pigs in what were becoming dense urban settlements during the later and post-medieval periods is well attested in the historical sources, which include a wide range of narratives following themes of urban pigs as public nuisances [78–80]. These stories about the trials of humans and pigs living in close proximity serve to highlight not only the challenges pigs posed to public order, but also to health. Urbanization and the husbandry of animals in urban settings is thought to have been a major factor in the pace and nature of development of zoonoses [81–83]. In that context, it is worth noting that, to the extent that our data show that pigs in Ireland began living in closer quarters to humans by the early medieval, the risk they presented with respect to the development and communication of zoonotic diseases may have antecedents reaching at least that far back. At the same time, we can also recognize the reverse implication, that of increased risk for zooanthroponosis that humans would pose to pigs when living in closer quarters [84].

While beyond the scope of the present study, it would also be worth considering these data in comparative contexts with Britain, much of which, unlike Ireland, came under Roman governance [85]. The arrival of Roman influence in Britain, occurring *ca* 350 years before the end of the Irish Iron Age, brought forms of settlement centralization [86] that, within our interpretive framework, could have led to more animal-protein-focused pig diets and could therefore be marked in the collective isotopic compositions of British post-Roman-occupation fauna. We recommend caution, however, when transposing Irish patterns to Britain and Europe. It is possible that pig husbandry in Ireland, and the implications this has for wider understanding of Irish settlement and farming habitats, unfolded in ways that were unique to the island. For this reason, factors influencing the ways that pigs were husbanded in, say, England could cumulatively result in pigs being raised closer to human settlements earlier in time than in Ireland. While numerous studies have examined archaeological pig isotopic compositions in England, Scotland and Wales [87–93], none have done so on the scale and from the baseline-corrected perspective that we have here. This means that the broadscale patterns we have observed cannot be readily compared.

### 4.4. Perceiving pigs: paragons or pariahs?

Our data suggest that the typical ways in which people interacted with pigs would have changed over time. During prehistory, when pigs lived at some remove from areas of dense human population, the day-to-day practical knowledge of pig management (i.e. their habits, ecology and biology), including the direct experience required to *know* pigs as individuals (i.e. their group dynamics and personalities [94]; see also [1]), may have been concentrated in the minds of relatively few specialized swine herders and others supporting that role. By contrast, while medieval and later swine herders would have maintained this role, during the pannage season at least [12], and the close connection with pigs that it entails, the shift to pigs living in closer association with people would have provided more exposure, including opportunities for day-to-day interaction and observation between pigs and people from a larger cross section of society. In other words, more people would have been forming more of their understanding of pigs—both abstractly as social and biological creatures and, in cases of closer sustained interaction, as individuals—based on firsthand experience.

While it is well beyond the scope of this study to unpack these themes in detail, the macroscale trends in the amount of day-to-day interaction between pigs and people revealed by our data offer novel opportunities to think about trends in the occurrence and abundance of meaningful two-way human–pig relationships over time. Here, isotopic data can contribute to interpretations of the past that do not centre on the human experience and, rather, allow us to consider how the agency of pigs would have influenced both animal and human decisions. Theoretical approaches that attempt to comprehend animals as persons, or as agents with a capacity to influence (rather than unidirectionally be influenced by) people [94] are firmly rooted in the ‘Animal Turn’ within the humanities [95,96]. While these approaches hold considerable promise for advancing the archaeology of human–animal relations, they require knowledge about direct, sustained interactions between humans and animals (a prerequisite for a genuine two-way exchange between persons [97,98]). This makes these approaches challenging to contextualize because nuanced evidence for sustained relationships between people and animals (even domestic livestock) in the archaeological past can be rare (e.g. much zooarchaeological data, for instance, reflect animal deaths rather than animal lives (although, see [99,100])). They are also typically, perhaps necessarily, focused on interactions located at the scale of the individual or small group. In this context, the approach we have used here, involving large-scale aggregation of *δ*^15^N data (contextualized with supplementary *δ*^13^C data) as proxy for proximity to humans, may offer a useful strategy for framing such narratives at a different, larger scale (for work at a similar scale, see [101]).

## 5. Conclusion

This is the first study to offer a detailed later-Holocene-scale picture of pig husbandry. While commendable work in numerous other studies has offered diverse insights into human–pig interactions, including studies that are both spatially and temporally extensive [102–111], our study has benefited from a synergy among key contextual elements—a robust herbivore baseline for correcting *δ*^15^N shifts, large sample sizes, and a relatively simple interpretive context from an isotope ecology perspective. These assets allow our interpretations to home in on specific changes in human–animal relationships, changes that imply significant turning points in areas of wider archaeological interest beyond husbandry, including settlement patterns, landscape management and economic structures. Our data show that for more than 4000 years, pigs were husbanded in ways that limited the kinds of foods available to them and mimicked the natural diet of their mainly herbivorous wild ancestors. During the early medieval that pattern changed and pigs, on average, began eating considerably greater amounts of animal protein. With respect to the prehistoric period, we suggest that these patterns could reflect a confluence of two factors that offer viable explanations: settlement patterns and the richness of pig habitats at locations removed from denser human populations. We suggest that change in land ownership and the consequent change in farming organization may have been a stronger driver. Furthermore, with respect to the medieval and post-medieval periods, new opportunities, including human-generated, animal-protein-rich food waste and the increasing availability of byproducts from dairy and other forms of agricultural and industrial processing, became a cornerstone of pig husbandry. We would like to emphasize that the simplified categories we have used here—i.e. pigs being more-or-less herbivorous versus strongly omnivorous—are almost certainly masking a great deal of complexity in the lived experience of people and pigs through time. In that context, we acknowledge that, both before and after the transition we have observed, there were likely manifold distinct ways people raised and related to pigs.

It is also worth pointing out some of the many potential avenues of future work that could help to unpack these patterns. At a basic level, we believe that further work exploring *δ*^13^C and *δ*^15^N in both pigs and baseline fauna would help to refine spatiotemporal dimensions of the transition from herbivory to omnivory. In that context, exceptions to the broad trends that we have observed will likely be identified. For instance, larger-scale husbandry operations that fed pigs using brewing and baking byproducts, as attested to in the historical record, would systematically generate pig remains with less animal-protein-influenced isotopic compositions. Such a scenario would produce livestock with lower *δ*^15^N and higher *δ*^13^C values, and could, for instance, account for a small number of pigs from Viking Age settlements, in both Ireland [46] and England [87], that show more herbivorous diets. Likewise, more refined approaches to estimating trophic position, such as *δ*^15^N analyses of single amino acids [112], could also offer more detail about variation in animal-protein consumption among prehistoric pigs. While more costly and therefore often applied on smaller scales, such techniques can generate data for establishing both baseline and trophic level from a single pig sample and can therefore offer interpretations at the individual level, compared to the population level to which our interpretations are necessarily limited. We would also suggest further work exploring trends in animal health and pathology through time. For instance, changes in pig husbandry, involving living in closer proximity to humans, could have left osteological markers in the form of trends in the abundance and kinds of pathologies preserved in time series of animal remains [81,113,114]. Changes in diet could also have been recorded in the calculus and metabolites preserved on teeth and in bones [115,116]. Against all these potential future avenues, however, this study highlights the importance of scale and the value of integrating larger quantities of data from broad spatial and temporal cross sections of archaeology.

Lastly, it is worth reflecting on our findings in the context of discussion on connections between Ireland’s earliest historical narratives and late prehistory (for a review, see [117]). There has been much debate about the value of transposing information from ethnographic and historical processes to earlier time periods for the purpose of interpreting archaeological patterns. In that context, it is interesting to consider that the two patterns for pig diets noted in the early medieval period literature—husbandry based in part on human-provisioned animal protein, on the one hand, and on woodland pannage, on the other—are not uniformly represented in the Neolithic–Early Bronze Age, Middle–Late Bronze Age or even most comparatively recent, Iron Age, past. While our data show a consistent importance of pannage extending from early prehistory through to the historic past, the emphasis on animal protein in medieval pig diets, and its absence in earlier periods, clearly illustrated the value of exercising caution when transposing historical processes to earlier times.

## 6. Methods

### 6.1. Sample

Our samples were collected for a project examining baseline *δ*^15^N variation throughout the Irish Holocene and a subset of the data presented here were published in a paper exploring that theme [43]. We have since been able to analyse a larger number of pigs from many sites and, by applying more stringent quality control metrics and considering both *δ*^15^N and *δ*^13^C from these new and previously published data [43,46,48,49,118], we are now able to offer interpretations focusing specifically on pigs.

Samples come from 38 sites (see electronic supplementary material, table S1) dating from the Neolithic to the post-medieval periods and spanning much of the island (figure 1). Our chronological framework for grouping samples is Neolithic–Early Bronze Age, *ca* 4000 BC to *ca* 1500 BC (*n* = 27); Middle–Late Bronze Age, *ca* 1500 BC to *ca* 500 BC (*n* = 40); Iron Age, *ca* 500 BC to *ca* AD 400 (*n* = 86); early medieval period, *ca* AD 400 to *ca* AD 1100 (*n* = 46); later medieval period, *ca* AD 1100 to *ca* AD 1550 (*n* = 58); and post-medieval/early modern period, *ca* AD 1550 to *ca* AD 1900 (*n* = 45). Samples were identified and recorded using standard zooarchaeological criteria [119], and young juveniles were excluded on the basis of either fusion, tooth eruption and wear, or size, as appropriate.

### 6.2. Collagen extraction and isotopic analyses

Collagen extractions followed two protocols, both modified from Longin [120]. Samples with a ‘SUBC’ prefix (*n* = 203) were demineralized in 0.5 M hydrochloric acid (HCl), then neutralized in Type 1 water (resistivity = 18 MΩ cm) and refluxed in a 10^−3^ HCl (pH3) solution in an oven at *ca* 65−70°C for 36–48 h. Gelatinized samples were then processed through 45−90 μm Ezee filters and 30 kDa molecular weight cut-off filters (ultrafilters) and then frozen and lyophilized. Samples with ‘IUBC’ prefix (*n* = 99) followed the same protocol with two changes: (i) Ezee and ultrafilters were not used; and (ii) a sodium hydroxide (NaOH) pre-treatment step (i.e. samples were soaked in a 0.1 M NaOH solution in an ultrasonic bath for 15 min cycles until solution remained clear) was performed after demineralization but before refluxing. We recognize that these two methods may not be equally effective at removing humic acids, the main potential contaminants for bone collagen, and therefore systematic *δ*^13^C differences could occur between samples treated with each method (for a review, see [53]). However, we apply the strictest possible quality control criteria, including conservative C : N [53] as well as standard carbon (>13.8%) and nitrogen (>4.0%) concentration metrics [121]. With respect to C : N, we have applied the conservative criteria, which were modelled to ensure measured bone collagen *δ*^13^C values deemed viable are within −0.5‰ of their biogenic *δ*^13^C value. Virtually all samples fell in the ‘<−20.00‰’ *δ*^13^C category, which allows for associated C : N values of no greater than 3.50 under the conservative criteria. By contrast, liberal criteria are less stringent and tolerate humic acid contamination-induced *δ*^13^C alterations of up to −1.0‰. As noted in §3, these criteria could technically be applied to two pig samples that showed evidence of marine diets (see electronic supplementary material, figure S3). These two samples produced *δ*^13^C values falling in the ‘<−20.00‰’ and ‘−19.99 to −18.00’ *δ*^13^C categories, which allow for associated C : N values of no greater than 3.70 and 3.55, respectively. In the context of these quality control metrics, we note that regardless of whether the two methods used for collagen extraction are equally effective at purifying collagen extracts (i.e. mainly removing humic acids), application of the conservative C : N criteria will ensure that any data used (regardless of what extraction protocol was applied) will be comparable, with minimal influence from contamination.

Isotopic compositions were measured on 0.5 mg subsamples of collagen using an elemental analyser coupled to an isotope ratio mass spectrometer in the Archaeology Chemistry Laboratory at the University of British Columbia. Measurements for 36% of samples were replicated. Isotopic compositions were calibrated using a two-point calibration curve anchored to international standards and accuracy and precision were monitored using a variety of check standards. Known and long-term observed isotopic compositions and s.d. for calibration and check standards are presented in electronic supplementary material, table S2. Standard deviations for calibration standards from all analytical sessions are presented in electronic supplementary material, table S3. Means and s.d. for check standards and sample replicates are shown in electronic supplementary material, tables S4 and S5, respectively. For *δ*^13^C and *δ*^15^N: systematic errors [*μ*_(bias)_] were ±0.11‰ and ± .15‰, respectively; random errors [*μ*R_(w)_] were ±0.13‰ and ±0.20‰, respectively; and standard uncertainties were ±0.16‰ and ±0.25‰, respectively [122].

### 6.3. Baseline adjustments

Previous research has shown that Irish herbivore *δ*^15^N values have increased over the Holocene, evidencing significant shifts in *δ*^15^N baseline variation occurring at the plant–soil level [43]. This phenomenon complicates the interpretation of potential patterns in higher trophic level animals, including omnivores such as pigs, because *δ*^15^N shifts in non-herbivorous animals could be linked to variation both in *δ*^15^N baseline and animal protein consumption. To obviate this issue while interpreting temporal patterns in pig *δ*^15^N values, we take two approaches.

First, the significance of changing levels of pig omnivory through time was evaluated by sequentially comparing baseline-corrected mean *δ*^15^N between adjacent time periods. For each comparison, the latter temporal groups’ *δ*^15^N values were adjusted according to the baseline offset observed between groups of cattle from those respective time periods. For instance, to establish the extent to which the change in pig *δ*^15^N between, say, the early medieval and the later medieval periods could be driven by omnivory rather than by fluctuations in baseline *δ*^15^N, we adjusted the isotopic composition of the later medieval pigs by the difference in mean *δ*^15^N (−0.7‰) that we had observed between cattle from the early medieval and later medieval periods. For a list of groups compared, see electronic supplementary material, table S9. We selected cattle for this purpose because they offer, by far, the largest published dataset of *δ*^15^N and *δ*^13^C values (also used in other comparison, see below), including *n* = 344 samples with associated quality control metrics meeting conservative C : N criteria [53].

Second, we aggregated isotopic data from across large numbers of sites for each time period. This offers a ‘zoomed out’ view of trends that is less likely to be impacted by site-specific processes, which may be more prone to reflect localized and otherwise unusual husbandry practices. To help further confirm that site-level patterns are in line with overall trends, where sample sizes permitted (i.e. for sites with more than five samples per taxon, see below), we performed site-level statistical comparisons to verify that these matched patterns observed at the more spatially encompassing time-period level.

The importance of mast in pig diets, which is ^13^C enriched relative to most other woodland foods in Ireland, was established in part through comparisons between cattle (which cannot eat large amounts of mast) and pigs (which will preferentially eat mast). For more detail, see §§2–4. Some of these comparisons require adjustments to account for the influence of a minor trophic enrichment factor (TEF) for *δ*^13^C (*ca* 0.5‰ per trophic level (TL) as compared with *ca* 3.6‰ for *δ*^15^N [34]). Specifically, historical pig temporal group *δ*^13^C means were adjusted according to TL (electronic supplementary material, table S10; figures 6–8 panels (*a*)). TL for pigs (TL_pig_) was established by subtracting the mean *δ*^15^N for cattle from the mean *δ*^15^N for pigs in each time period and then dividing this number by the expected *δ*^15^N TEF (i.e. 3.6‰). The amount of adjustment for pig *δ*^13^C was then calculated by multiplying TL_pig_ by the TEF for *δ*^13^C (i.e. 0.5‰). This adjustment was then subtracted from pig *δ*^13^C values for respective time-period groups. Comparisons were then performed using recalculated mean pig *δ*^13^C for the early, later and post-medieval periods versus cattle from the early medieval period.

### 6.4. Other interpretive considerations

While our dataset is large by archaeological standards, spatial coverage per time period does not provide an opportunity explore geographical variation in detail. Nonetheless, we use the opportunity created by our comparison of site-level and time period-level trends (see above) to consider whether regional patterns emerge. We also note that previous work on Irish archaeological pig and cattle isotopic compositions, aggregated at the island-wide scale, indicates that broadscale patterns reflect temporal changes rather than geographical factors [23,43]. Moreover, we note that these previous analyses [23,43] showed no correlation between trends in archaeological faunal isotopic compositions (either *δ*^13^C or *δ*^15^N) and known climate oscillations across the Irish Holocene [123–125], suggesting that, at this island-wide, aggregated scale, changing environmental conditions may be less important, relative to human impacts, in driving isotopic variation.

We are aware that interspecific differences in isotopic diet-to-collagen spacing can occur and, as such, there could be systematic variation in the way that cattle and pigs eating the same diet express *δ*^13^C and *δ*^15^N values. With respect to *δ*^15^N, while a wider range of inter-specific variation has been observed in diet-to-collagen spacing (also known as trophic enrichment factors), evaluation of the extent to which these could be applicable to our interpretations is complicated. This is due to the diverse range of variables (e.g. nutritional status, diet composition, growth rate) which can, at both the inter-specific/intra-digestive-physiology and the intra-specific levels, dramatically impact diet-to-collagen spacing size [126–128]. These factors cannot be known for animals from the archaeological past. Despite the wide range and complexity of this variation, broadscale syntheses of observations across a taxonomically diverse range of species suggest among mammals diet-to-collagen spacing for *δ*^15^N is on average similar across taxa [34]. We note that across intra-site interspecific comparisons in this study (table 2), pigs provide examples of cases where average *δ*^15^N values are higher or lower than those of cattle, suggesting any systematic difference, if present at all, would likely be very small.

With respect to *δ*^13^C, systematic differences have been documented between diet and collagen relative to other tissues from the same animals [129,130], which are thought to be linked with differences in respective species’ digestive physiologies [131]. Studies examining cattle and pigs independently, as well as other taxa, have shown that, in addition to digestive physiology, diverse variables, again including factors such as nutritional status and diet quality, can dramatically impact diet-to-collagen spacing for *δ*^13^C between animals for the same species [126,132,133]. Other recent work exploring spacing between enamel (bioapatite) and diet among animals with differing digestive physiologies has suggested that animals with ruminant-fermenter digestive systems, such as cattle, have *δ*^13^C values that are *ca* 1‰ higher than those with non-coprophagous hindgut-fermenter digestive systems, such as pigs [71]. However, bone collagen and bioapatite are constructed and maintained using materials from distinctive biomolecular pools within the body, making the enamel–collagen tissue spacing notoriously difficult to characterize [131]. To concretely resolve the question of whether, and under what conditions, there may be a consistent and meaningful interspecific diet-to-collagen offset in *δ*^13^C between cattle and pigs, in-depth controlled feeding experiments are needed to quantify the impacts of different diets, nutritional statuses and environmental conditions on bone collagen *δ*^13^C in cattle and pigs raised under the same conditions. In summary, as with *δ*^15^N, because intraspecific differences in diet–collagen *δ*^13^C are contingent on basic factors like diet quality, which are unknowable for archaeological animals, it is not possible to correct for the potential effects of digestive physiology on *δ*^13^C. In that context, we note that we are not aware of research suggesting that pigs and cattle should have a pronounced difference (e.g. >1‰) in *δ*^13^C diet–collagen offset.

Considering the above, while we recognize that it is possible that under certain conditions, cattle and pig bone collagen isotopic compositions may reflect diet in slightly different ways, we do not attempt to apply a correction when comparing cattle and pig *δ*^15^N and *δ*^13^C.

### 6.5. Statistics

Statistical comparisons were conducted in PAST version 4.13 [134]. We tested for normality of sample group distributions using Shapiro–Wilk tests. Where groups were not normally distributed, a Mann–Whitney *U* test was used to compare group means. In cases where compared groups were normally distributed, a Levene’s test was used to evaluate homogeneity of variance. Where variance was equal between groups, a Student’s *t*-test was used to compare means. Where variance was not equal between groups, a Welch’s *t*-test was used to compare means. Effect size was determined by Cohen’s *d* for parametric tests (i.e. Student’s *t* and Welch’s *t*) and Vargha–Delaney *A* was used for non-parametric tests (i.e. Mann–Whitney *U*).

## Data Availability

All data are included in the paper and the electronic supplementary material [135].

## Footnotes

^1^
Human-generated animal protein may include scraps and waste from human meals and or byproducts from hunting, agricultural activities or industrial processes or human or domestic animal excrement. From the perspective of impacts on pig bone collagen δ^15^N, all of these potential foods falling under the umbrella of animal protein are interchangeable, serving to drive up δ^15^N.

## References

[1] Kreiner J. 2020 Legions of pigs in the early medieval west. New Haven, CT: Yale University Press. (10.12987/yale/9780300246292.001.0001)

[2] Albarella U, Dobney K, Ervynck A, Rowley-Conwy P. 2007 Pigs and humans: 10,000 years of interaction. Oxford, UK: Oxford University Press.

[3] Albarella U, Payne S. 2005 Neolithic pigs from Durrington Walls, Wiltshire, England: a biometrical database. J. Archaeol. Sci. **32**, 589–599. (10.1016/j.jas.2004.11.008)

[4] Payne S, Bull G. 1988 Components of variation in measurements to distinguish wild from domestic pig remain. Archaeozoologica II **1–2**, 27–66.

[5] Putman RJ. 2008 Pigs. In Mammals of the British Isles: handbook (eds S Harris, D Yalden), pp. 563–564. Southampton, UK: Mammal Society.

[6] Copado F, de Aluja AS, Mayagoitia L, Galindo F. 2004 The behaviour of free ranging pigs in the Mexican tropics and its relationships with human faeces consumption. Appl. Anim. Behav. Sci. **88**, 243–252. (10.1016/j.applanim.2004.03.013)

[7] Albarella U, Manconi F, Albarella U, Dobney K, Ervynck A, Rowley-Conwy P. 2007 Ethnoarchaeology of pig husbandry in Sardinia and Corsica. In Pigs and humans: 10,000 years of interaction. Oxford, UK: Oxford University Press. (10.1093/oso/9780199207046.003.0027)

[8] Silver IA. 1963 The aging of domestic animals. In Science in archaeology: a survey of progress and research (eds D Brothwell, E Higgs), pp. 250–268. London, UK: Basic Books.

[9] Danfaer A, Strathe A. 2012 Quantitative and physiological aspects of pig growth. In Nutrition and physiology of the pig (eds KEB Knudsen, NJ Kjeldsen, HD Poulsen, BB Jensen). Copenhagen, Denmark: SEGES Danish Pig Research Centre.

[10] Beglane F. 2016 The faunal remains. In The Bective Abbey project: archaeological excavations 2009–12 (eds G Stout, M Stout), pp. 106–153. Dublin, Ireland: Wordwell.

[11] Parsons JJ. 1962 The acorn-hog economy of the oak woodlands of southwestern Spain. Geogr. Rev. **52**, 211. (10.2307/212957)

[12] Kelly F. 1997 Early Irish farming. Dublin, Ireland: School of Celtic Studies.

[13] McCormick F. 2007 Mammal bone studies from prehistoric Irish sites. In Environmental archaeology in Ireland (eds EM Murphy, NJ Whitehouse), pp. 77–101. Oxford, UK: Oxbow.

[14] McCormick F, Murray E. 2007 Excavations at Knowth, vol. 3: Knowth and the zooarchaeology of early Christian Ireland. In Royal Irish Academy monographs in archaeology. Dublin, Ireland: Royal Irish Academy.

[15] Pigière F, McCormick F, Olet L, Moore D, O’Carroll F, Smyth J. 2022 More than meat? Examining cattle slaughter, feasting and deposition in later 4th millennium BC Atlantic Europe: a case study from Kilshane, Ireland. J. Archaeol. Sci. **41**, 103312. (10.1016/j.jasrep.2021.103312)

[16] McCormick F. 1991 The effect of the Anglo-Norman settlement on Ireland’s wild and domesticated fauna. In Animal use and culture change (eds PJ Crabtree, K Ryan), pp. 41–52. Philadelphia, PA: University Museum of Archaeology and Anthropology, University of Pennsylvania.

[17] McCormick F, Murray E. 2017 The zooarchaeology of medieval Ireland. In The Oxford handbook of zooarchaeology, pp. 195–213. Oxford, UK: Oxford University Press. (10.1093/oxfordhb/9780199686476.013.15)

[18] Beglane F. 2009 Meat and craft in medieval and post-medieval Trim. In Uncovering medieval Trim: archaeological excavations in and around Trim, Co. Meath (eds M Potterton, M Seaver), pp. 346–370. Dublin, Ireland: Four Courts Press.

[19] Beglane F. 2009 Long pig’s feet from Iron Age Trim. In Uncovering medieval Trim: archaeological excavations in and around Trim, Co. Meath (eds M Potterton, M Seaver), pp. 57–71. Dublin, Ireland: Four Courts Press.

[20] Beglane F. 2023 The fat of the land. In Food provisioning in complex societies: zooarchaeological perspectives (eds L Atici, BS Arbuckle), pp. 71–91. Denver, CO: University Press of Colorado. (10.5876/9781646422562.c004)

[21] Szpak P. 2022 Why zooarchaeology needs stable isotope analysis. In Isotope research in zooarchaeology, pp. 248–270. Boca Raton, FL: University Press of Florida. (10.2307/j.ctv2zx9q60.15)

[22] Guiry EJ, Gaulton BC. 2016 Inferring human behaviors from isotopic analyses of rat diet: a critical review and historical application. J. Archaeol. Method Theory **23**, 399–426. (10.1007/s10816-015-9248-9)

[23] Guiry E, Beglane F, Szpak P, McCormick F, Teeter MA, Cheung C, Richards MP. 2023 Changing human-cattle relationships in Ireland: a 6000-year isotopic perspective. Antiquity **97**, 1436–1452. (10.15184/aqy.2023.163)

[24] Tieszen LL. 1991 Natural variations in the carbon isotope values of plants: implications for archaeology, ecology, and paleoecology. J. Archaeol. Sci. **18**, 227–248. (10.1016/0305-4403(91)90063-u)

[25] Bonafini M, Pellegrini M, Ditchfield P, Pollard AM. 2013 Investigation of the ‘canopy effect’ in the isotope ecology of temperate woodlands. J. Archaeol. Sci. **40**, 3926–3935. (10.1016/j.jas.2013.03.028)

[26] van der Merwe NJ, Medina E. 1991 The canopy effect, carbon isotope ratios and foodwebs in Amazonia. J. Archaeol. Sci. **18**, 249–259. (10.1016/0305-4403(91)90064-v)

[27] Guiry E, Orchard TJ, Needs-Howarth S, Szpak P. 2021 Isotopic evidence for garden hunting and resource depression in the late woodland of northeastern North America. Am. Antiq. **86**, 90–110. (10.1017/aaq.2020.86)

[28] Guiry EJ, Orchard TJ, Royle TCA, Cheung C, Yang DY. 2020 Dietary plasticity and the extinction of the passenger pigeon (Ectopistes migratorius). Quat. Sci. Rev. **233**, 106225. (10.1016/j.quascirev.2020.106225)

[29] Cernusak LA *et al*. 2009 Why are non-photosynthetic tissues generally ¹³C enriched compared with leaves in C₃ plants? Review and synthesis of current hypotheses. Funct. Plant Biol. **36**, 199–213. (10.1071/FP08216) 32688639

[30] Styring AK, Jirdén E, Lagerås P, Larsson M, Sjöström A, Ljung K. 2024 Carbon isotope values of hazelnut shells: a new proxy for canopy density. Front. Environ. Archaeol. **3**, 1351411. (10.3389/fearc.2024.1351411)

[31] Sarà M, Sarà G. 2007 Trophic habits of Muscardinus avellanarius (Mammalia Gliridae) as revealed by multiple stable isotope analysis. Ethol. Ecol. Evol. **19**, 215–223. (10.1080/08927014.2007.9522563)

[32] Selva N, Hobson KA, Cortés-Avizanda A, Zalewski A, Donázar JA. 2012 Mast pulses shape trophic interactions between fluctuating rodent populations in a primeval forest. PLoS ONE **7**, e51267. (10.1371/journal.pone.0051267) 23251475 PMC3519590

[33] DeNiro MJ, Epstein S. 1978 Influence of diet on the distribution of carbon isotopes in animals. Geochim. Cosmochim. Acta **42**, 495–506. (10.1016/0016-7037(78)90199-0)

[34] Caut S, Angulo E, Courchamp F. 2009 Variation in discrimination factors (Δ^15^N and Δ^13^C): the effect of diet isotopic values and applications for diet reconstruction. J. Appl. Ecol. **46**, 443–453. (10.1111/j.1365-2664.2009.01620.x)

[35] Guiry E. 2019 Complexities of stable carbon and nitrogen isotope biogeochemistry in ancient freshwater ecosystems: implications for the study of past subsistence and environmental change. Front. Ecol. Evol. **7**, 313. (10.3389/fevo.2019.00313)

[36] Fan R, Morozumi T, Maximov TC, Sugimoto A. 2018 Effect of floods on the δ^13^C values in plant leaves: a study of willows in northeastern Siberia . PeerJ **6**, e5374. (10.7717/peerj.5374) 30258705 PMC6151259

[37] Chisholm BS, Nelson DE, Schwarcz HP. 1982 Stable-carbon isotope ratios as a measure of marine versus terrestrial protein in ancient diets. Science **216**, 1131–1132. (10.1126/science.216.4550.1131) 17808502

[38] Losey RJ, Guiry E, Nomokonova T, Gusev AV, Szpak P. 2020 Storing fish? A dog’s isotopic biography provides insight into Iron Age food preservation strategies in the Russian Arctic. Archaeol. Anthropol. Sci. **12**. (10.1007/s12520-020-01166-3) PMC7410107 32831958

[39] Hamilton J, Hedges REM, Robinson M. 2009 Rooting for pigfruit: pig feeding in Neolithic and Iron Age Britain compared. Antiquity **83**, 998–1011. (10.1017/s0003598x00099300)

[40] Szpak P. 2014 Complexities of nitrogen isotope biogeochemistry in plant-soil systems: implications for the study of ancient agricultural and animal management practices. Front. Plant Sci. **5**, 288. (10.3389/fpls.2014.00288) 25002865 PMC4066317

[41] Katzenberg MA, Pfeiffer S. 1995 Nitrogen isotope evidence for weaning age in a nineteenth century Canadian skeletal sample. In Bodies of evidence: reconstructing history through skeletal analysis (ed. A Grauer), pp. 221–235. New York, NY: Wiley-Liss.

[42] Minagawa M, Wada E. 1984 Stepwise enrichment of ¹⁵N along food chains: further evidence and the relation between δ¹⁵N and animal age. Geochim. Cosmochim. Acta **48**, 1135–1140. (10.1016/0016-7037(84)90204-7)

[43] Guiry E, Beglane F, Szpak P, Schulting R, McCormick F, Richards MP. 2018 Anthropogenic changes to the Holocene nitrogen cycle in Ireland. Sci. Adv. **4**, s9383. (10.1126/sciadv.aas9383) PMC6007156 29928695

[44] Guiry EJ, Orchard TJ, Needs-Howarth S, Szpak P. 2022 Freshwater wetland-driven variation in sulfur isotope compositions: implications for human paleodiet and ecological research. Front. Ecol. Evol. **10**, 953042. (10.3389/fevo.2022.953042)

[45] Guiry E, Noël S, Fowler J. 2021 Archaeological herbivore δ¹³C and δ³⁴S provide a marker for saltmarsh use and new insights into the process of ¹⁵N-enrichment in coastal plants. J. Archaeol. Sci. **125**, 105295. (10.1016/j.jas.2020.105295)

[46] Knudson KJ, O’Donnabhain B, Carver C, Cleland R, Price TD. 2012 Migration and Viking Dublin: paleomobility and paleodiet through isotopic analyses. J. Archaeol. Sci. **39**, 308–320. (10.1016/j.jas.2011.09.014)

[47] Ryan SE, Reynard LM, Crowley QG, Snoeck C, Tuross N. 2018 Early medieval reliance on the land and the local: an integrated multi-isotope study (⁸⁷Sr/⁸⁶Sr, δ¹⁸O, δ¹³C, δ¹⁵N) of diet and migration in Co. Meath, Ireland. J. Archaeol. Sci. **98**, 59–71. (10.1016/j.jas.2018.08.002)

[48] McKenzie CJ, Murphy EM, Guiry E, Donnelly CJ, Beglane F. 2020 Diet in medieval Gaelic Ireland: a multiproxy study of the human remains from Ballyhanna, Co. Donegal. J. Archaeol. Sci. **121**, 105203. (10.1016/j.jas.2020.105203)

[49] Madgwick R, Grimes V, Lamb AL, Nederbragt AJ, Evans JA, McCormick F. 2019 Feasting and mobility in Iron Age Ireland: multi-isotope analysis reveals the vast catchment of Navan Fort, Ulster. Sci. Rep. **9**, 19792. (10.1038/s41598-019-55671-0) 31874966 PMC6930251

[50] Woodman P, Mccarthy M, Monaghan N. 1997 The Irish Quaternary fauna project. Quat. Sci. Rev. **16**, 129–159. (10.1016/s0277-3791(96)00037-6)

[51] Jensen P, Recén B. 1989 When to wean—observations from free-ranging domestic pigs. Appl. Anim. Behav. Sci. **23**, 49–60. (10.1016/0168-1591(89)90006-3)

[52] Ó Baoill R. 2001 2001:309—Greencastle, Down. Excavations.ie: database of Irish excavation reports.

[53] Guiry EJ, Szpak P. 2021 Improved quality control criteria for stable carbon and nitrogen isotope measurements of ancient bone collagen. J. Archaeol. Sci. **132**, 105416. (10.1016/j.jas.2021.105416)

[54] Kyriazakis I, Emmans GC, Whittemore CT. 1990 Diet selection in pigs: choices made by growing pigs given foods of different protein concentrations. Anim. Sci. **51**, 189–199. (10.1017/s0003356100005298)

[55] Rose SP, Kyriazakis I. 1991 Diet selection of pigs and poultry. Proc. Nutr. Soc. **50**, 87–98. (10.1079/pns19910014) 1881934

[56] Westendorf ML. 2000 Food waste as animal feed: an introduction. In Food waste to animal feed, pp. 3–16. Ames, IA: Iowa State University Press. (10.1002/9780470290217)

[57] Whitehouse NJ *et al*. 2014 Neolithic agriculture on the European western frontier: the boom and bust of early farming in Ireland. J. Archaeol. Sci. **51**, 181–205. (10.1016/j.jas.2013.08.009)

[58] Cooney G. 2012 Reading a landscape manuscript: a review of progress in prehistoric settlement studies in Ireland. In A history of settlement in Ireland, pp. 17–65. London, UK: Routledge. (10.4324/9780203025192-8)

[59] Szabó P. 2013 Rethinking pannage: historical interactions between oak and swine. In Trees, forested landscapes and grazing animals (ed. ID Rotherham), pp. 51–61. London, UK: Routledge.

[60] Wealleans AL. 2013 Such as pigs eat: the rise and fall of the pannage pig in the UK. J. Sci. Food Agric. **93**, 2076–2083. (10.1002/jsfa.6145) 23553313

[61] Lamond E. 1890 Walter of Henley’s husbandry: together with an anonymous husbandry, seneschaucie and Robert Grosseteste’s rules. London, UK: Longmans, Green and Co.

[62] Plunkett G. 2007 Pollen analysis and archaeology in Ireland. In Environmental archaeology in Ireland (ed. NJ Whitehouse), pp. 221–240. Oxford, UK: Oxbow Books.

[63] Plunkett G. 2020 A palynological perspective on ‘An archaeology of Ireland for the information age’. Emania **25**, 39–43.

[64] Kelly F. 1988 A guide to early Irish law. Dublin, Ireland: Dublin Institute for Advanced Studies.

[65] Mytum HC. 1992 The origins of early Christian Ireland. London, UK: Routledge.

[66] Becker K, Armit I, Swindles G, Stanley M. 2017 New perspectives on the Irish Iron Age: the impact of NRA development on our understanding of later prehistory. In Stories of Ireland’s past (eds M Stanley, R Swan, A O’Sullivan), pp. 85–100. Dublin, Ireland: Transport Infrastructure Ireland.

[67] Kenward H, Hall A, Allison E, Carrott J. 2011 Environment, activity and living conditions at Deer Park Farms: evidence from plant and invertebrate remains. In Deer Park Farms: the excavation of a raised rath in the Glenarm valley, Co. Antrim. Northern Ireland archaeological monographs (eds C Lynn, JA McDowell), pp. 498–547. Belfast, UK: Stationery Office.

[68] McCormick F. 1995 Cows, ringforts and the origins of early Christian Ireland. Emania **13**, 33–37.

[69] Beglane F. 2018 Forests and chases in medieval Ireland, 1169–c .1399. J. Hist. Geogr. **59**, 90–99. (10.1016/j.jhg.2017.11.002)

[70] Beglane F. 2015 Anglo-Norman parks in medieval Ireland. Dublin, Ireland: Four Courts Press.

[71] Cerling TE, Bernasconi SM, Hofstetter LS, Jaggi M, Wyss F, Rudolf von Rohr C, Clauss M. 2021 CH₄/CO₂ ratios and carbon isotope enrichment between diet and breath in herbivorous mammals. Front. Ecol. Evol. **9**, 638568. (10.3389/fevo.2021.638568)

[72] Guiry E, Beglane F, Carlin N, Orton D, Teeter M, Szpak P. Submitted. Pigs, pannage, and the solstice: isotopic insights from prehistoric feasting at Newgrange.

[73] González-Barrio R, Truchado P, García-Villalba R, Hervás G, Frutos P, Espín JC, Tomás-Barberán FA. 2012 Metabolism of oak leaf ellagitannins and urolithin production in beef cattle. J. Agric. Food Chem. **60**, 3068–3077. (10.1021/jf300718k) 22375726

[74] Cedervall A, Johansson HE, Jönsson L. 1973 Acorn poisoning in cattle. Nord. Vet. Med. **25**, 639–644. 4768237

[75] McCormick F. 2008 The decline of the cow: agricultural and settlement change in early medieval Ireland. Peritia **20**, 209–224. (10.1484/j.peri.3.632)

[76] Thomas R. 2009 Bones of contention: why later post-medieval faunal assemblages in Britain matter. In Crossing paths or sharing tracks?, pp. 133–148. Woodbridge, UK: Boydell and Brewer. (10.1515/9781846157103-017)

[77] Thomas R, Fothergill BT. 2014 Foreword. Anthropozoologica **49**, 11–18. (10.5252/az2014n1a01)

[78] Cantwell I. 2001 Anthropozoological relationships in late medieval Dublin. Dublin Hist. Rec. **54**, 73–80.

[79] Adelman J. 2020 Civilised by beasts. Manchester, UK: Manchester University Press.

[80] Nugent J. 2009 The human snout: pigs, priests, and peasants in the parlor. Senses Soc. **4**, 283–301. (10.2752/174589209x12464528171851)

[81] Bartosiewicz L. 2022 Ancient zoonoses. In Zoonoses: infections affecting humans and animals, pp. 1–23. Dordrecht, The Netherlands: Springer. (10.1007/978-3-030-85877-3_54-1)

[82] Tanga C, Remigio M, Viciano J. 2022 Transmission of zoonotic diseases in the daily life of ancient Pompeii and Herculaneum (79 CE, Italy): a review of animal–human–environment interactions through biological, historical and archaeological sources. Animals **12**, 213. (10.3390/ani12020213) 35049834 PMC8773449

[83] Uhl EW, Thomas R. 2022 Uncovering tales of transmission: an integrated palaeopathological perspective on the evolution of shared human and animal pathogens. In Palaeopathology and evolutionary medicine, pp. 317–349. Oxford, UK: Oxford University Press. (10.1093/oso/9780198849711.003.0017)

[84] Messenger AM, Barnes AN, Gray GC. 2014 Reverse zoonotic disease transmission (zooanthroponosis): a systematic review of seldom-documented human biological threats to animals. PLoS ONE **9**, e89055. (10.1371/journal.pone.0089055) 24586500 PMC3938448

[85] Johnston E. 2017 Ireland in late antiquity. Stud. Late Antiq. **1**, 107–123. (10.1525/sla.2017.1.2.107)

[86] Redfern RC. 2020 Changing people, changing settlements? A perspective on urbanism from Roman Britain. In Bioarchaeology and social theory: the bioarchaeology of urbanization, pp. 25–47. Dordrecht, The Netherlands: Springer. (10.1007/978-3-030-53417-2_2)

[87] Hammond C, O’Connor T. 2013 Pig diet in medieval York: carbon and nitrogen stable isotopes. Archaeol. Anthropol. Sci. **5**, 123–127. (10.1007/s12520-013-0123-x)

[88] Jones JR, Mulville J. 2016 Isotopic and zooarchaeological approaches towards understanding aquatic resource use in human economies and animal management in the prehistoric Scottish North Atlantic Islands. J. Archaeol. Sci. **6**, 665–677. (10.1016/j.jasrep.2015.08.019)

[89] Madgwick R, Mulville J, Stevens RE. 2012 Diversity in foddering strategy and herd management in late Bronze Age Britain: an isotopic investigation of pigs and other fauna from two midden sites. Environ. Archaeol. **17**, 126–140. (10.1179/1461410312z.00000000011)

[90] Schulting RJ *et al*. 2019 The ups & downs of Iron Age animal management on the Oxfordshire Ridgeway, south-central England: a multi-isotope approach. J. Archaeol. Sci. **101**, 199–212. (10.1016/j.jas.2018.09.006)

[91] Hamilton J, Thomas R. 2012 Pannage, pulses and pigs: isotopic and zooarchaeological evidence for changing pig management practices in later medieval England. Mediev. Archaeol. **56**, 234–259. (10.1179/0076609712z.0000000008)

[92] Millard AR, Jimenez‐Cano NG, Lebrasseur O, Sakai Y. 2013 Isotopic investigation of animal husbandry in the Welsh and English periods at Dryslwyn Castle, Carmarthenshire, Wales. Int. J. Osteoarchaeol. **23**, 640–650. (10.1002/oa.1292)

[93] Stevens RE, Lightfoot E, Hamilton J, Cunliffe BW, Hedges REM. 2013 One for the master and one for the dame: stable isotope investigations of Iron Age animal husbandry in the Danebury environs. Archaeol. Anthropol. Sci. **5**, 95–109. (10.1007/s12520-012-0114-3)

[94] Knight J. 2005 Animals in person: cultural perspectives on human-animal intimacies. Oxford, UK: Berg.

[95] Ritvo H. 2007 On the animal turn. Daedalus **136**, 118–122. (10.1162/daed.2007.136.4.118)

[96] Overton NJ, Hamilakis Y. 2013 A manifesto for a social zooarchaeology. Swans and other beings in the Mesolithic. Archaeol. Dialogues **20**, 111–136. (10.1017/s1380203813000159)

[97] Dwyer PD, Minnegal M. 2020 Person, place or pig: animal attachments and human transactions in New Guinea. In Animals in person (ed. J Knight), pp. 37–60. London, UK: Routledge. (10.4324/9781003135883-3)

[98] Theodossopoulos D. 2020 Care, order and usefulness: the context of the human–animal relationship in a Greek island community. In Animals in person (ed. J Knight), pp. 15–35. London, UK: Routledge. (10.4324/9781003135883-2)

[99] Argent G. 2010 Do the clothes make the horse? Relationality, roles and statuses in Iron Age Inner Asia. World Archaeol. **42**, 157–174. (10.1080/00438241003672633)

[100] Szpak P, Millaire JF, White CD, Longstaffe FJ. 2014 Small scale camelid husbandry on the north coast of Peru (Virú Valley): insight from stable isotope analysis. J. Anth. Arch. **36**, 110–129.

[101] Orton D. 2010 Both subject and object: herding, inalienability and sentient property in prehistory. World Archaeol. **42**, 188–200. (10.1080/00438241003672773)

[102] Frémondeau D, De Cupere B, Evin A, Van Neer W. 2017 Diversity in pig husbandry from the Classical-Hellenistic to the Byzantine periods: an integrated dental analysis of Düzen Tepe and Sagalassos assemblages (Turkey). J. Archaeol. Sci. **11**, 38–52. (10.1016/j.jasrep.2016.11.030)

[103] Aiken M. 2019 Pig husbandry at Kastro Kallithea: an isotopic study of pig husbandry in Hellenistic Thessaly. Master thesis, University of Alberta, Canada.

[104] Kennedy JR, Guiry EJ. 2023 Exploring railroad impacts on meat trade: an isotopic investigation of meat sourcing and animal husbandry at Chinese Diaspora sites in the American West. Int. J. Hist. Archaeol. **27**, 393–423. (10.1007/s10761-022-00663-6)

[105] Madgwick R, Lamb AL, Sloane H, Nederbragt AJ, Albarella U, Pearson MP, Evans JA. 2019 Multi-isotope analysis reveals that feasts in the Stonehenge environs and across Wessex drew people and animals from throughout Britain. Sci. Adv. **5**, u6078. (10.1126/sciadv.aau6078) PMC6415963 30891495

[106] Halley DJ, Rosvold J. 2014 Stable isotope analysis and variation in medieval domestic pig husbandry practices in northwest Europe: absence of evidence for a purely herbivorous diet. J. Archaeol. Sci. **49**, 1–5. (10.1016/j.jas.2014.04.006)

[107] Guiry E, Jones BM, deFrance S, Bruseth JE, Durst J, Richards MP. 2018 Animal husbandry and colonial adaptive behavior: isotopic insights from the La Belle shipwreck fauna. Hist. Archaeol. **52**, 684–699. (10.1007/s41636-018-0142-7)

[108] Balasse M, Cucchi T, Evin A, Bălăşescu A, Frémondeau D, Horard-Herbin MP. 2018 Wild game or farm animal? Tracking human-pig relationships in ancient times through stable isotope analysis. In Hybrid communities, pp. 81–96. London, UK: Routledge. (10.4324/9781315179988-5)

[109] Cucchi T, Dai L, Balasse M, Zhao C, Gao J, Hu Y, Yuan J, Vigne JD. 2016 Social complexification and pig (Sus scrofa) husbandry in ancient China: a combined geometric morphometric and isotopic approach. PLoS ONE **11**, e0158523. (10.1371/journal.pone.0158523) 27384523 PMC4934769

[110] Vaiglova P, Reid REB, Lightfoot E, Pilaar Birch SE, Wang H, Chen G, Li S, Jones M, Liu X. 2021 Localized management of non-indigenous animal domesticates in Northwestern China during the Bronze Age. Sci. Rep. **11**, 15764. (10.1038/s41598-021-95233-x) 34344976 PMC8333310

[111] Guiry EJ, Staniforth M, Nehlich O, Grimes V, Smith C, Harpley B, Noël S, Richards MP. 2015 Tracing historical animal husbandry, meat trade, and food provisioning: a multi-isotopic approach to the analysis of shipwreck faunal remains from the William Salthouse, Port Phillip, Australia. J. Archaeol. Sci. **1**, 21–28. (10.1016/j.jasrep.2014.10.001)

[112] McMahon KW, Newsome SD. 2019 Amino acid isotope analysis: a new frontier in studies of animal migration and foraging ecology. In Tracking animal migration with stable isotopes (eds KA Hobson, LI Wassenaar), pp. 173–190, 2nd edn. Waltham, MA: Academic Press. (10.1016/B978-0-12-814723-8.00007-6)

[113] Thomas R. 2019 Nonhuman animal paleopathology—are we so different? In Ortner’s identification of pathological conditions in human skeletal remains, pp. 809–822. San Diego, CA: Elsevier. (10.1016/b978-0-12-809738-0.00023-5)

[114] Bendrey R, Martin D. 2022 Zoonotic diseases: new directions in human-animal pathology. Int. J. Osteoarchaeol. **32**, 548–552. (10.1002/oa.2975) 33821116 PMC8014110

[115] Alldritt I *et al*. 2019 Metabolomics reveals diet-derived plant polyphenols accumulate in physiological bone. Sci. Rep. **9**, 8047. (10.1038/s41598-019-44390-1) 31142795 PMC6541599

[116] Weber S, Price MD. 2016 What the pig ate: a microbotanical study of pig dental calculus from 10th–3rd millennium BC northern Mesopotamia. J. Archaeol. Sci. **6**, 819–827. (10.1016/j.jasrep.2015.11.016)

[117] Gleeson P. 2020 Archaeology and myth in early medieval Europe: making the gods of early Ireland. Mediev. Archaeol. **64**, 65–93. (10.1080/00766097.2020.1754646)

[118] Schulting R. 2014 The dating of Poulnabrone. In Poulnabrone, Co. Clare: excavation of an early Neolithic portal tomb (ed. A Lynch), pp. 93–113. Dublin, Ireland: Stationery Office.

[119] Davis SJ. 1992 A rapid method for recording information about mammal bones from archaeological sites. Ancient Monuments Laboratory Report 19/92.

[120] Longin R. 1971 New method of collagen extraction for radiocarbon dating. Nature **230**, 241–242. (10.1038/230241a0) 4926713

[121] Ambrose SH. 1990 Preparation and characterization of bone and tooth collagen for isotopic analysis. J. Archaeol. Sci. **17**, 431–451. (10.1016/0305-4403(90)90007-r)

[122] Szpak P, Metcalfe JZ, Macdonald RA. 2017 Best practices for calibrating and reporting stable isotope measurements in archaeology. J. Archaeol. Sci. **13**, 609–616. (10.1016/j.jasrep.2017.05.007)

[123] McClung LC, Plunkett G. 2020 Cultural change and the climate record in final prehistoric and early medieval Ireland. Proc. R. Ir. Acad. **120C**, 129–158. (10.1353/ria.2020.0014)

[124] Kerr TR, Swindles GT, Plunkett G. 2009 Making hay while the sun shines? Socio-economic change, cereal production and climatic deterioration in Early Medieval Ireland. J. Archaeol. Sci. **36**, 2868–2874. (10.1016/j.jas.2009.09.015)

[125] Swindles GT *et al*. 2013 Centennial-scale climate change in Ireland during the Holocene. Earth Sci. Rev. **126**, 300–320. (10.1016/j.earscirev.2013.08.012)

[126] Warinner C, Tuross N. 2009 Alkaline cooking and stable isotope tissue-diet spacing in swine: archaeological implications. J. Archaeol. Sci. **36**, 1690–1697. (10.1016/j.jas.2009.03.034)

[127] Sponheimer M *et al*. 2003 Nitrogen isotopes in mammalian herbivores: hair δ^15^N values from a controlled feeding study. Int. J. Osteoarchaeol. **13**, 80–87. (10.1002/oa.655)

[128] Warinner C, Tuross N. 2010 Brief communication. Tissue isotopic enrichment associated with growth depression in a pig: implications for archaeology and ecology. Am. J. Phys. Anthropol. **141**, 486–493. (10.1002/ajpa.21222) 20052664

[129] van der Merwe NJ, Vogel JC. 1978 13C Content of human collagen as a measure of prehistoric diet in woodland North America. Nature **276**, 815–816. (10.1038/276815a0) 364321

[130] Ambrose SH, Norr L. 1993 Experimental evidence for the relationship of the carbon isotope ratios of whole diet and dietary protein to those of bone collagen and carbonate. In Prehistoric human bone, pp. 1–37. Cham, Switzerland: Springer. (10.1007/978-3-662-02894-0_1)

[131] Hedges REM. 2003 On bone collagen–apatite‐carbonate isotopic relationships. Int. J. Osteoarchaeol. **13**, 66–79. (10.1002/oa.660)

[132] Sponheimer M *et al*. 2003 An experimental study of carbon-isotope fractionation between diet, hair, and feces of mammalian herbivores. Can. J. Zool. **81**, 871–876. (10.1139/z03-066)

[133] Froehle AW, Kellner CM, Schoeninger MJ. 2010 FOCUS: effect of diet and protein source on carbon stable isotope ratios in collagen: follow up to Warinner and Tuross (2009). J. Archaeol. Sci. **37**, 2662–2670. (10.1016/j.jas.2010.06.003)

[134] Hammer Ø, Harper DA, Ryan PD. 2001 PAST: paleontological statistics software package for education and data analysis. Palaeontol. Electron. **4**, 9.

[135] Guiry E, Beglane F, Tourigny E, McCormick F, Richards MP. 2024 Supplementary material from: Pigs, people, and proximity: a 6000-year isotopic record of pig management in Ireland. Figshare. (10.6084/m9.figshare.c.7577661) PMC11793965 39911886

